# Mis-targeting of the mitochondrial protein LIPT2 leads to apoptotic cell death

**DOI:** 10.1371/journal.pone.0179591

**Published:** 2017-06-19

**Authors:** Emanuele Bernardinelli, Roberta Costa, Giada Scantamburlo, Janet To, Rossana Morabito, Charity Nofziger, Carolina Doerrier, Gerhard Krumschnabel, Markus Paulmichl, Silvia Dossena

**Affiliations:** 1 Institute of Pharmacology and Toxicology, Paracelsus Medical University, Salzburg, Austria; 2 School of Biological Sciences, Nanyang Technological University, Singapore, Singapore; 3 Department of Chemical, Biological, Pharmaceutical and Environmental Sciences, University of Messina, Messina, Italy; 4 Oroboros Instruments, Innsbruck, Austria; 5 Center for Health and Bioresources, Austrian Institute of Technology, Vienna, Austria; University of PECS Medical School, HUNGARY

## Abstract

Lipoyl(Octanoyl) Transferase 2 (LIPT2) is a protein involved in the post-translational modification of key energy metabolism enzymes in humans. Defects of lipoic acid synthesis and transfer start to emerge as causes of fatal or severe early-onset disease. We show that the first 31 amino acids of the N-terminus of LIPT2 represent a mitochondrial targeting sequence and inhibition of the transit of LIPT2 to the mitochondrion results in apoptotic cell death associated with activation of the apoptotic volume decrease (AVD) current in normotonic conditions, as well as over-activation of the swelling-activated chloride current (IClswell), mitochondrial membrane potential collapse, caspase-3 cleavage and nuclear DNA fragmentation. The findings presented here may help elucidate the molecular mechanisms underlying derangements of lipoic acid biosynthesis.

## Introduction

Lipoylation is a post-translational modification involving five lipoate-dependent enzymes which catalyze essential redox reactions in humans. Two of these enzymes (α-ketoglutarate dehydrogenase and pyruvate dehydrogenase, PDH) play an essential role in mitochondrial energy metabolism, and three (branched-chain ketoacid dehydrogenase, 2-oxoadipate dehydrogenase, and the glycine cleavage system, GCS) are involved in amino acid metabolism. The first four enzymes are collectively denoted as 2-oxoacid dehydrogenases. The lipoate-dependent enzymes are multicomplex proteins, and lipoylation involves the E2 subunit/E3 binding protein and the H-protein of 2-oxoacid dehydrogenases and GCS, respectively [1].

**Fig 1:**
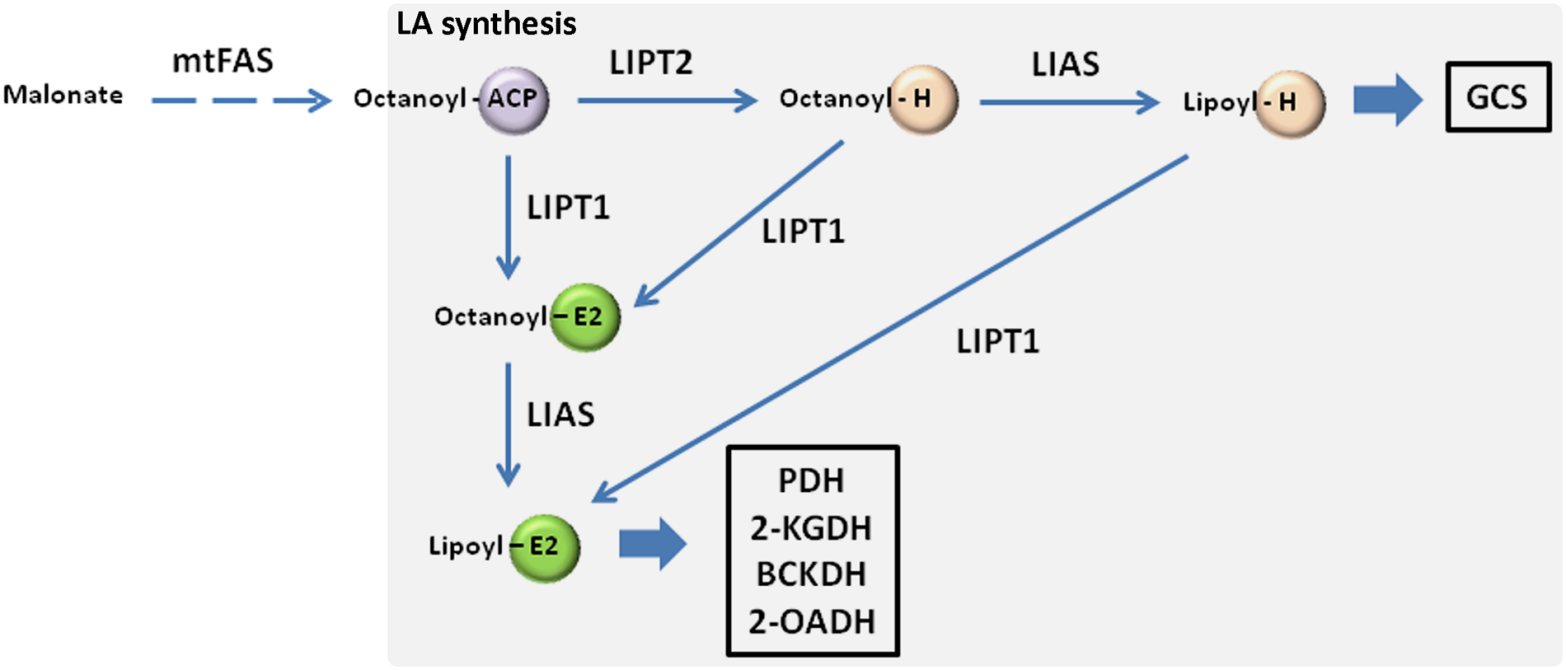
Lipoic acid biosynthesis. mtFAS generates octanoyl-ACP, that enters the lipoic acid biosynthesis pathway. The octanoyl moiety is then transferred from ACP to H or E2 proteins. Subsequently, insertion of two sulfur atoms occurs on the octanoyl moiety to generate lipoylated H or E2 proteins. 2-KGDH, α-ketoglutarate dehydrogenase; 2-OADH, 2-oxoadipate dehydrogenase; ACP, acyl carrier protein; BCKDH, branched-chain ketoacid dehydrogenase; GCS, glycine cleavage system; LA, lipoic acid; LIAS, lipoic acid synthetase; LIPT1, lipoyl(octanoyl) transferase 1; LIPT2, lipoyl(octanoyl) transferase 2; mtFAS, mitochondrial fatty acidynthesis; PDH, pyruvate dehydrogenase.

Lipoic acid (6,8 dithiooctanoic acid) is a small hydrophobic molecule consisting of eight carbons and two sulfhydryl groups, first identified in association with PDH [2]. While being well characterized in *E*. *coli*, the biosynthesis of lipoic acid in eukaryotes is not completely understood and relies on recent studies in yeast. In eukaryotic cells, the activity of lipoate-dependent enzymes appears to rely exclusively on *de novo* intramitochondrial lipoic acid synthesis [3]. In the mitochondrial fatty acid synthesis (mtFAS) pathway, octanoic acid—the precursor of lipoic acid—is synthesized from malonate and conjugated to an acyl carrier protein (ACP). Then, octanoic acid is transferred to the H protein of GCS system *via* action of lipoyl(octanoyl) transferase 2 (LIP2 in yeast; LIPT2, putative, in humans) (Fig 1). In the reaction, the free carboxyl group of octanoic acid is attached *via* an amide linkage to the epsilon-amino group of a conserved lysine residue within a conserved lipoyl domain. Octanoylated H protein is the substrate for insertion of two sulfur atoms at C-6 and C-8 positions to obtain lipoylated H protein in a reaction catalyzed by the iron-sulphur (Fe-S) cluster protein lipoic acid synthetase (LIP5 in yeast; LIAS in humans). An additional enzyme (LIP3 in yeast; LIPT1 in humans) catalyzes the transfer of octanoic/lipoic acid to the E2 subunits of the 2-oxoacid dehydrogenase complexes [4,5] (Fig 1).

The relevance of lipoic acid biosynthesis in mammalians was elucidated by the use of cell lines [6] or knockout mice [7] in which the expression of key components of this pathway was compromised. These studies evidenced that mtFAS and lipoylation of mitochondrial proteins are tightly related and essential for mitochondrial function, cell survival and energy utilization. Lipoic acid biosynthesis defects were reported to be associated with human disease for the first time in 2011, when mutations in genes encoding for Fe-S cluster proteins (LIAS, MIM 60703) [8] or proteins involved in their biogenesis (NFU1, MIM 608100; and BOLA3, MIM 613183) [9,10] were identified as responsible for the patients’ phenotype. Later, mutations affecting lipoic acid transfer pathway (LIPT1, MIM 610284) have also been described [5,11]. Patients with lipoic acid deficiency present common, early-onset clinical features such as psychomotor retardation, leukoencephalopathy and hypotonia; pulmonary hypertension and cardiomyopathy may also be present. Abnormalities of biochemical parameters include altered levels of organic acids (lactate, 2-ketoglutarate) and glycine, and PDH deficiency. The gravity of symptoms often results in fatalities [5]. Very recently, mutations involving the *LIPT2* gene (c.89T>C; c.377T>G) were identified by exome sequencing in a 8-year-old boy with encephalopathy, axial hypotonia and spasticity associated to mitochondrial respiratory chain deficiency in brain, impaired leucine metabolism and severely decreased PDH activity [12].

A detailed understanding of structure, function, biogenesis and trafficking of proteins involved in the synthesis and transfer of lipoic acid is essential to understand the molecular mechanisms of diseases resulting from lipoylation defects. Among the proteins involved in lipoic acid turnover, very little is known about LIPT2. LIPT2 function has been inferred by sequence similarity with the homologues genes in *E*. *coli* (*lipB*) [13] and *S*. *cerevisiae* (*LIP2*) [14]. Also, no experimental evidence to confirm the identity of the predicted mitochondrion transit peptide has been published. Curiously, a high throughput assay based on the mammalian two-hybrid method identified a direct protein interaction between the mouse homologue of LIPT2 and B-myc [15], a nuclear regulator of transcription and cellular proliferation [16,17]. In addition, LIPT2 protein expression has been referred to as cytoplasmic in most tissues and, on a cellular level, mainly localized in vesicles or the centrosome (www.proteinatlas.org, accessed on the 30^th^ of January 2017). Therefore, we aimed to verify the expression and subcellular localization of LIPT2 in human cell lines, as well as the sequence of its mitochondrial targeting peptide. Here we show that amino acids 1–31 of LIPT2 target the protein to the mitochondrion. In addition, incorrect trafficking of LIPT2 to its physiological compartment leads to apoptotic cell death.

## Materials and methods

### Plasmids

The ORF coding for lipoyl(octanoyl) transferase 2 (LIPT2; NCBI sequence ID: NP_001138341.1) was cloned from HEK Blue cDNA into the pET3-His vector [18] with the following primers: forward, 5’ GAC GCA ACG GTG GGC ACG A 3’, and reverse, 5’ TGG CTC AGA GCT CAT GGT ATG TCC 3’, and then subcloned into the XhoI and BamHI restriction sites of the bicistronic mammalian expression vector pIRES2 EGFP (Clontech). The use of vectors bearing the internal ribosome entry site (IRES) allows for the simultaneous expression of two individual proteins—LIPT2 and the enhanced green fluorescent protein (EGFP)—from a single bicistronic mRNA without the production of fusion proteins [19]. The single transfected cells can be individuated optically by detecting the fluorescent light emitted by EGFP (excitation maximum, 488 nm; emission maximum, 507 nm). When this vector was used for electrophysiology experiments, control experiments were conducted in cells transfected with the pIRES2-EGFP-EGFP vector, where an additional EGFP coding sequence was cloned into the pIRES2-EGFP expression vector [20].

The ORF of LIPT2 was further subcloned into the XhoI and BamHI restriction sites of pEYFPC1 and pEYFPN1 mammalian expression vectors (Clontech), in frame with the ORF of the enhanced yellow fluorescent protein (EYFP). These constructs encode for LIPT2 with EYFP fused to its N (EYFP-LIPT2) or C-terminus (LIPT2-EYFP), respectively.

A construct encoding for LIPT2 void of the putative transit peptide to mitochondrion and with EYFP fused to its C-terminus (ΔmitotagLIPT2-EYFP) was obtained by amplification of the sequence coding for amino acids 32–231 of LIPT2 using the pET3-His vector as the template and the following primers: forward, 5’ CAT TAC TCG AGA TGG CAG AGC CCG GCA TTG 3’, and reverse, 5’ ATA TAG GAT CCA CGT TGG GGC TGT CC 3’, and cloning the PCR product into the XhoI and BamHI restriction sites of pEYFPN1 vector.

To obtain EYFP with the LIPT2 putative mitochondrion transit peptide fused to its N-terminus (mitotag-EYFP), the sequence coding for the LIPT2 putative mitochondrion transit peptide (amino acids 1–31) was amplified by PCR using the pET3-His vector as the template and the following primers: forward, 5’ ACC AGG CTC GAG AGA TGC GGC AAC CC 3’, and reverse, 5’ ATA CCT GGA TCC GCC TGC AGC CGC CGC 3’, and cloned into the XhoI an BamHI restriction sites of pEYFPN1 vector, in frame with the ORF of EYFP.

To obtain the mitotag-ΔATG-EYFP construct, the initiation of translation (START) codon (ATG) of the EYFP ORF was deleted from the sequence coding mitotag-EYFP with the QuikChange^®^ site-directed mutagenesis kit (Stratagene) according to the manufacturer’s protocol and the following primers: forward, 5’ CCA CCG GTC GCC ACC GTG AGC AAG GGC GAG G 3’, and reverse, 5’ CCT CGC CCT TGC TCA CGG TGG CGA CCG GTG G 3’.

To obtain a construct encoding for the red fluorescent protein dsRed with the LIPT2 putative mitochondrion transit peptide fused to the N-terminus (mitotag-dsRed), the ORF coding for dsRed was amplified using the pIRES2 dsRed Express vector (Clontech) as the template ad the following primers: forward, 5’ AAG ACT GGA TCC TGC CTC ATC AGA AGA CGT CA 3’ and reverse, 5’ AGA GTC GCG GCC GCT ACA GGA ACA GGT GGT GG 3’, and subcloned into the BamHI and NotI restriction sites of the mitotag-ΔATG-EYFP construct.

All plasmid inserts were sequenced prior to use in experiments (Microsynth AG, Switzerland).

### Cell culture and transient transfection

Human embryonic kidney (HEK) 293 Phoenix [21] and HeLa (human cervical adenocarcinoma, CCL-2, directly obtained from American Type Cell Culture Collection) cells were cultured in Minimum Essential Eagle Medium (Sigma-Aldrich, Austria) supplemented with 10% fetal bovine serum (Lonza), 2 mM L-glutamine, 100 U/ml penicillin, 100 μg/ml streptomycin and 1 mM pyruvic acid (sodium salt). The cells were maintained at 37°C, 5% CO_2_, 95% air and 100% humidity. Subcultures were routinely established every second to third day by seeding the cells into 100 mm diameter Petri dishes following trypsin/ethylenediaminetetraacetic acid (EDTA) treatment.

For transfection, cells were seeded into 6-well plates and grown overnight to ~50% confluence. HeLa cells were transfected with 1.5 μg of plasmid DNA and 3 μl of Metafectene Pro (Biontex). HEK 293 Phoenix cells were transfected with 1–3 μg of plasmid DNA using the calcium phosphate co-precipitation method. Transfection medium was replaced with fresh medium 8 hours post-transfection. Transfection efficiency was ~35% and ~20% in HEK 293 Phoenix and HeLa cells, respectively.

### Mitochondrial extraction

Mitochondrial isolation was performed using the Qproteome Mitochondria Isolation Kit (Qiagen). HEK 293 Phoenix cells were seeded into two 15 cm diameter Petri dishes and cultured for 4 days. Cells (40 x 10^6^) were washed 3 times with 20 ml ice-cold phosphate buffer saline (PBS) and then scraped in 10 ml PBS on ice. Cellular proteins were fractionated into cytosolic, microsomal and mitochondrial fractions according to manufacturer’s instruction. Shortly, following centrifugation at 500 *x g* for 10 min at 4°C, the supernatant was removed and the cell pellet was washed with 1 ml 0.9% (w/v) ice-cold NaCl solution. Cells were then centrifuged again as described previously. For the following steps, all samples and buffers were incubated on ice and all centrifugation steps were performed at 4°C. After discarding the supernatant, the cell pellet was resuspended in 2 ml Lysis Buffer supplemented with Halt protease inhibitor cocktail (HPIC, Thermo) to a final concentration of 1X and incubated at 4°C for 10 min with end-over-end mixing. The lysate was centrifuged at 1000 *x g* for 10 min, and the supernatant (cytosolic fraction) was carefully removed and saved for later use. The cell pellet was resuspended in 1 ml Disruption Buffer supplemented with HPIC to a final concentration of 1X. Cell disruption was completed with 70 strokes in a 5 ml teflon homogenizer. The lysate was transferred to a 2 ml tube and centrifuged at 1000 *x g* for 10 min to remove nuclei, cell debris and unbroken cells. The supernatant was transferred to a clean 1.5 ml tube and centrifuged at 6000 *x g* for 10 min to obtain the mitochondrial pellet. The supernatant (microsomal fraction) was carefully removed and saved for later use. The mitochondrial pellet was processed according to the high-purity preparation procedure following the manufacturer’s instruction. Shortly, the mitochondrial pellet was resuspended in 750 μl Mitochondrial Purification Buffer. A separate 750 μl aliquot of Mitochondrial Purification Buffer was added to a clean 2 ml tube and carefully overlayed with 500 μl Disruption Buffer. The mitochondrial suspension was pipetted on top of the two phases and centrifuged at 14000 *x g* for 15 min. 1.5 ml of the supernatant were removed and the remaining solution was transferred to a new 2 ml tube. This solution was diluted with 1.5 ml Mitochondria Storage Buffer and centrifuged at 8000 *x g* for 10 min. This step was repeated until a visible pellet of mitochondria formed at the bottom of the tube. For western blotting, the mitochondrial pellet was solubilized in 100 μL 1X SDS-containing solubilization buffer (SSB) by vortexing for 1 min. Cytosolic and microsomal fractions were diluted 1:2 in 2X SSB. SSB (4X) contained 0.55 M SDS and 5.42 M glycerol in 0.5 M Trizma base, pH 6.8. SSB dilutions were obtained with water. Dithiothreitol was added fresh to 1X SSB to a final concentration of 1 mM. Protein concentration was determined with the DC protein assay (Biorad).

### Western blot

To obtain whole-cell lysates, 1–3 x 10^6^ cells were collected by centrifugation, washed in PBS and lysed in 100–200 μl of ice-cold lysis buffer (20 mM Tris-HCl pH 8, 150 mM NaCl, 1 mM EDTA, 1% NP40, 1X Halt Protease Inhibitor Cocktail, Thermo) on ice. Cell lysates were centrifuged at 16,000 *x g* at 4°C for 30 minutes. The supernatant was collected and stored at -80°C until use. Protein extracts (≥20 μg for whole-cell lysates, 20 μg for mitochondrial, microsomal and cytosolic fractions) were electrophoresed with constant voltage (120 V) on SDS-PAGE gels (10 or 14%). Proteins were then transferred overnight onto polyvinylidene fluoride (PVDF) membranes with constant amperage (0.25 mA). The membranes were blocked for 1 hour at room temperature in 5% nonfat dry milk diluted in Tris-buffered saline containing 0.01% Tween 20, pH 7.6 (TBST). Afterwards, PVDF membranes were incubated overnight at 4°C with primary antibodies diluted in TBST containing 5% nonfat dry milk, washed 3 times in TBST, incubated for 1 hour in the dark and at room temperature with the secondary antibody diluted in TBST containing 5% nonfat dry milk, washed again and kept in TBST. Immunocomplexes were visualized using the ODYSSEY infrared imaging system (LICOR) or the Chemidoc Doc^™^ MP imaging system (Biorad) following exposure to the Super Signal^®^ West Dura Extended Duration Substrate (Thermo). The rabbit anti-LIPT2 polyclonal antibody (HPA040249, 1:200 dilution), the mouse anti-α-tubulin (05–829, 1:1,000 dilution) and the mouse anti-ox-Phos-Complex IV (459600, 1:1,000 dilution) monoclonal antibodies were from SIGMA, Upstate and Invitrogen respectively. The rabbit anti-cytochrome c (#4272, 1:500 dilution), rabbit anti-β actin (#4967, 1:1,000 dilution) and rabbit anti-caspase-3 (#9662, 1:500 dilution) polyclonal antibodies and the rabbit anti-cleaved caspase-3 (#9664, 1:250 dilution) monoclonal antibody were from Cell Signaling. The rabbit anti-calreticulin (ab4, 1:1000 dilution), goat anti-GAPDH (JP_A00191-40, 1:1000 dilution), polyclonal antibodies and the rat anti-caspase-12 (sc-21747, 1:200 dilution) monoclonal antibody were from Abcam, GenScript and Santa Cruz, respectively. The anti-rabbit (926–32211, 1:10,000 dilution) and the anti-mouse (926–32210, 1:10,000 dilution) IRD-800-CW secondary antibodies were from LICOR. The goat anti-rat HRP-conjugated secondary antibody (AP136P, 1:10,000 dilution) was from Millipore.

### Patch clamp experiments

Transfected HEK 293 Phoenix cells were seeded on glass coverslips (diameter, 10 mm) contained in 30 mm diameter Petri dishes and grown overnight. Patch clamp experiments were performed 48 hours post-transfection. Single transfected cells were selected by fluorescence microscopy and voltage clamped using the whole-cell patch clamp technique as previously described [20,22–25]. Pipette (125 mM CsCl, 5 mM MgCl_2_, 11 mM EGTA, 2 mM ATP-Mg^++^, 10 mM HEPES, pH 7.2 adjusted with CsOH, osmotic pressure 300 mOsm/Kg_H2O_ adjusted with raffinose), hypertonic (125 mM NaCl, 2.5 mM CaCl_2_, 2.5 mM MgCl_2_, 10 mM HEPES, pH 7.4 adjusted with NaOH, osmotic pressure 360 mOsm/Kg_H2O_ adjusted with mannitol), isotonic (125 mM NaCl, 2.5 mM CaCl_2_, 2.5 mM MgCl_2_, 10 mM HEPES, pH 7.4 adjusted with NaOH, osmotic pressure 300 mOsm/Kg_H2O_ adjusted with mannitol) and hypotonic (125 mM NaCl, 2.5 mM CaCl_2_, 2.5 mM MgCl_2_, 10 mM HEPES, pH 7.4 adjusted with NaOH, osmotic pressure 260 mOsm/Kg_H2O_) bath solutions were designed to isolate chloride currents. The resistance of the glass pipettes filled with the pipette solution and immersed in the bath solution was 3 to 8 MΩ. Fast exchange of the hypertonic with the hypotonic bath solution was accomplished using a perfusion system with a flow rate of 5 ml/min and a bath volume of ∼300 μl. For data acquisition, an EPC-10 (HEKA Elektronik, Germany) amplifier controlled by a Macintosh computer running the Patch Master (HEKA Elektronik, Germany) software was used. Access resistance as well as fast and slow capacitance were compensated and monitored throughout the recordings. All current measurements were filtered at 5 kHz and digitized at 50 kHz. The cells were held at 0 mV, and step pulses of 400 ms duration were applied from 0 mV to +40 mV every 20 s to monitor current changes over time. To establish the current-to-voltage (I/V) relationship, step pulses of 500 ms duration were applied every 5 min from −120 mV to +100 mV in 20 mV increments from a holding potential of 0 mV. For data analysis, Fit Master (HEKA Elektronik, Germany) and Excel (Microsoft, USA) software were used. The current values (pA) were normalized to the membrane capacitance (pF) to obtain the current density (pA/pF), which is a measure of the current magnitude independent from the cell size. All experiments were carried out at room temperature.

### Colocalization experiments

For colocalization experiments, cells were transferred on round (3 cm diameter) glass slides 8–56 hours post-transfection and imaged 24–72 hours post-transfection.

To stain the mitochondria, living cells were washed thrice with Hank’s balanced salt solution (HBSS, Sigma-Aldrich), incubated for 15 minutes at room temperature with Mito Tracker Deep Red (M22426, Invitrogen Molecular Probes; 300 nM in HBSS) and washed again thrice with HBSS. Imaging was performed in HBSS at room temperature. EYFP was excited with the 514 nm line of the Argon laser and emission was detected in the 525–600 nm range; Mito Tracker Deep Red was excited at 633 nm (HeNe laser) and emission was detected in the 645–750 nm range.

**Fig 2:**
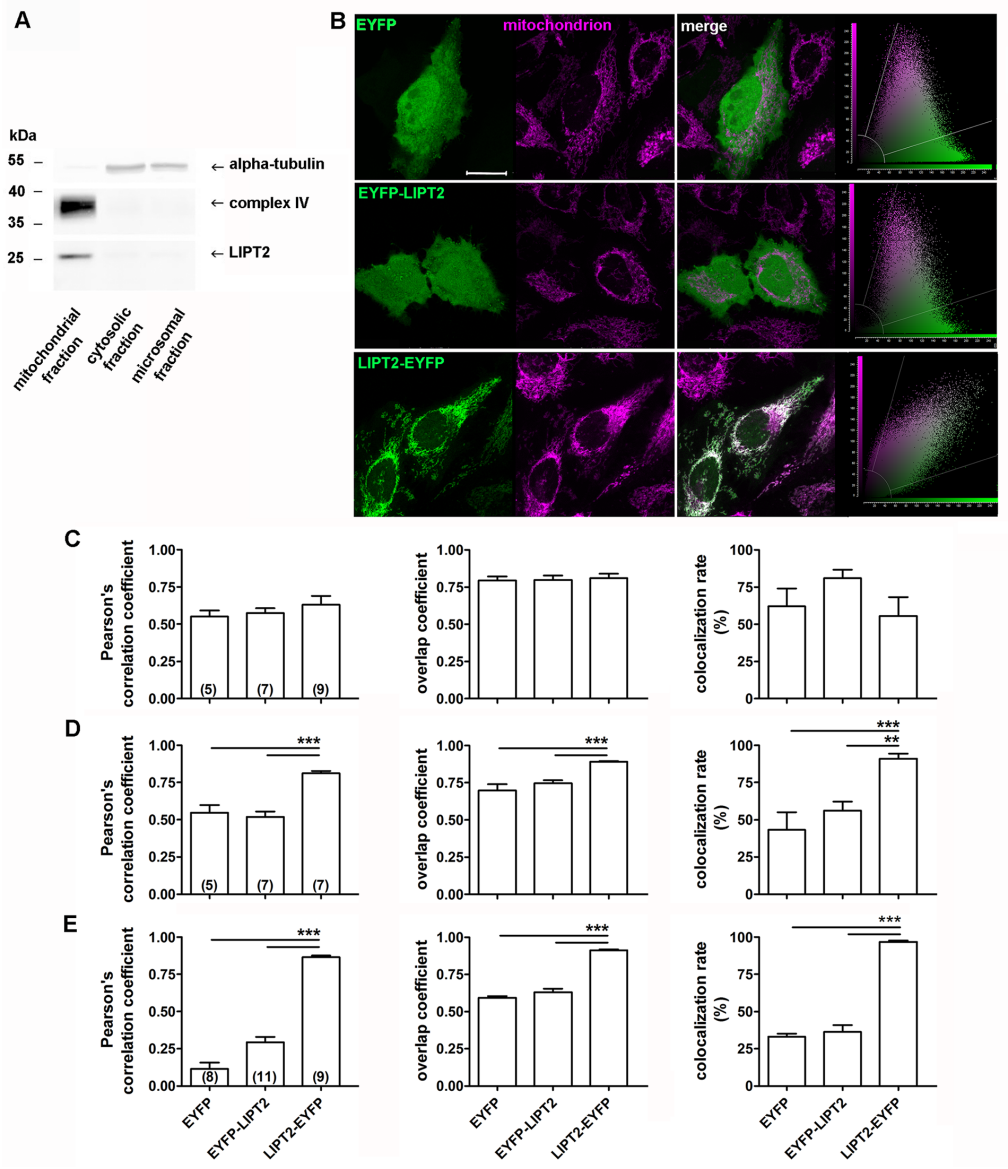
LIPT2 and LIPT2-EYFP target the mitochondrion. (A) Immunodetection of endogenous LIPT2 (~25 kDa) in mitochondrial, cytosolic and microsomal fractions obtained from HEK 293 Phoenix cellular proteins. Alpha-tubulin (55 kDa) and ox-Phos-complex IV (35–40 kDa) were used as markers of the non-mitochondrial (cytosolic and microsomal) and mitochondrial fractions, respectively. The image is representative of three independent experiments. (B) From left to right: fluorescent signal of EYFP (green) and the mitochondrial marker (magenta) in HeLa cells expressing EYFP, EYFP-LIPT2 or LIPT2-EYFP for 72 hours, corresponding merge image and scatter plot. Scale bar: 20 μm. Pearson’s correlation coefficient, overlap coefficient and co-localization rate (%) referred to the co-localization of EYFP, EYFP-LIPT2 or LIPT2-EYFP and the mitochondrion determined in HeLa cells (C), 24, (D), 48 and (E), 72 hours after transfection. (n) indicates the number of cells. **: p<0.01, ***: p<0.001, one-way ANOVA with Bonferroni’s post-test.

Imaging was performed by sequential acquisition with a Leica TCS SP5II AOBS confocal microscope (Leica Microsystems, Germany) equipped with a HCX PL APO 63x/1.20 Lambda blue water immersion objective and controlled by the LAS AF software (version 2.7.3.9723, Leica Microsystems). Colocalization was determined with the colocalization tool of LAS AF software. The presence of white pixels in the merge image and the distribution of pixels along the diagonal in the scatter plots (Figs 1 and 2) are indicative of co-localization between the two signals. Similarly, the absence of white pixels in the merge image and the distribution of pixels along the x and y axes in the scatter plots are indicative of an absence of co-localization between the two signals. Colocalization was quantified as the Pearson’s correlation coefficient [26] (that may range from -1 to +1 indicating complete spatial exclusion or co-localization of two fluorescent signals, respectively), the overlap coefficient and the colocalization rate (that may range from 0 to +1 indicating complete spatial exclusion or co-localization of two fluorescent signals, respectively). Localization of EYFP, a water-soluble protein with apparent homogeneous distribution within nucleus and cytosol, was taken as an indicator of an absence of preferential colocalization with the mitochondria.

### RNA interference

Small interfering (si) RNAs for knocking down the expression of LIPT2 (core sequence siRNA #1: 5’-GAA GUA AUG CCA CCU UUC CTT-3’; siRNA #2: 5’-GGA GAU CUA CAA GUG CAC ATT-3’; siRNA #3: 5’-GUA AUG CCA CCU UUC CUU GTT-3’) were designed with the siRNA Design Tool of Microsynth CH. HEK 293 Phoenix cells seeded into 30 mm diameter Petri dishes were transfected with 270–360 pmol of siRNAs and 18–24 μl of Metafectene SI (Biontex) following the manufacturer’s instruction. Control cells were transfected with the following negative control siRNA (Microsynth CH): 5’-GCA GCA CGA CUU CUU CAA GTT-3’. Functional (patch clamp) or expression (semiquantitative reverse transcription PCR) assays were performed 48–72 h after transfection.

### Semiquantitative reverse transcription-PCR

Extraction of total RNA from HEK 293 Phoenix cells transfected with LIPT2 siRNAs was performed with the All Prep DNA/RNA mini kit (Qiagen). One μg of total RNA was used for the reverse transcription reaction with the QuantiTect^®^ reverse transcription kit for cDNA synthesis with integrated removal of genomic DNA contamination (Qiagen). For detecting the LIPT2 transcript, the following primers were used: forward, 5’-AGC TGC TTT GCC ACC CGG TAC-3’; and reverse, 5’-GTG CCT TCC ACA GCG GAC TC-3’. The LIPT2 signal was normalized to the β-actin signal, detected by using the following primers: forward, 5’-GGC ATG GGT CAG AAG GAT TC-3’; and reverse, 5’-AGA GGC GTA CAG GGA TAG CAC-3’. These primers span an intron-exon boundary and would disclose an eventual contamination from genomic DNA as a band at 740 bp, which was not detected. Densitometric analysis was done with the ImageJ 1.46r software (Wayne Rasband, NIH, USA).

### DNA fragmentation assay

DNA fragmentation was detected *in situ* on cells seeded on poly-L-lysine-coated glass slides (12 mm diameter) with the Click-It^®^ Plus Terminal deoxynucleotidyl transferase dUTP nick end labeling (TUNEL) Assay (Molecular Probes Life Technologies) following the manufacturer’s instructions. Nuclei were stained with 0.1 μg/ml 4',6-diamidino-2-phenylindole (DAPI) in PBS for 10 minutes at room temperature. Then, slides were mounted in 90% glycerol/10% PBS, sealed and imaged with a Leica TCS SP5II AOBS confocal microscope equipped with a HCS PL APO CS 63x/1.4 oil immersion objective. DAPI was excited at 405 nm (diode laser) and emission was detected in the 445–490 nm range; Alexa Fluor^®^ 647 picolyl azide (TUNEL signal) was excited at 633 nm (HeNe laser) and emission was detected in the 645–750 nm range. To obtain the TUNEL signal normalized for the cell density, fluorescence intensity in the emission window of TUNEL was expressed as levels of gray, subtracted for the background fluorescence and normalized for the background-subtracted fluorescence intensity in the emission window of DAPI. Cells left untreated or treated overnight with 2 μM staurosporine were considered as the negative and the positive control, respectively. In the positive control, a TUNEL signal was detected in 100% of nuclei.

### Determination of mitochondrial membrane potential alterations on a single-cell level

To determine mitochondrial membrane potential alterations, cells were transferred on round (3 cm diameter) poly-L-lysine-coated glass slides 8–56 hours post-transfection and imaged 24–72 hours post-transfection.

To stain the mitochondria and nuclei, living cells were washed thrice with HBSS, incubated for 30 minutes at room temperature with 1 μg/ml Hoechst (Sigma Aldrich) in HBSS in the presence of 300 nM Mito Tracker Deep Red for the last 15 minutes and washed again thrice with HBSS. In control experiments, the mitochondrial membrane potential collapse was induced with the uncoupling agent carbonylcyanide-4-trifluoromethoxyphenylhydrazone (FCCP, Sigma Aldrich; 20μM) added to the staining solution. The vehicle of FCCP was dimethyl sulfoxide (DMSO). Imaging was performed by sequential acquisition in HBSS at room temperature with a Leica TCS SP5II AOBS confocal microscope equipped with a HCX PL APO 63×/1.20 Lambda blue water immersion objective. Hoechst was excited with the 405 nm line of a diode laser and emission was detected in the 430–470 nm range; EYFP was excited with the 514 nm line of the Argon laser and emission was detected in the 525–560 nm range; Mito Tracker Deep Red was excited at 633 nm (HeNe laser) and emission was detected in the 645–750 nm range. In control experiments, vehicle and FCCP-treated untransfected cells were used and Mito Tracker Deep Red signal was normalized for the cell density. For this, fluorescence intensity of Mito Tracker Deep Red determined in the whole imaging field was expressed as levels of gray, subtracted for the background fluorescence and normalized for the background-subtracted fluorescence intensity of Hoechst. Mitochondrial membrane potential alterations in cells transfected with LIPT2 constructs were determined on a single-cell level. For this, fluorescence intensity of Mito Tracker Deep Red was determined in a region of interest (ROI) within a transfected (EYFP expressing) cell, corrected for the background fluorescence and normalized for the background-subtracted fluorescence intensity of Mito Tracker Deep Red determined in a ROI of the same size within an adjacent untransfected cell.

### Salts and chemicals

All salts and chemicals used were of *per analysis* grade.

### Statistical analysis

All data were expressed as arithmetic means ± S.E.M. For statistical analysis, GraphPad Prism software (version 4.00 for Windows, GraphPad Software, San Diego, California, USA) was used. Significant differences between data sets were tested by one way Analysis of Variance (ANOVA) with Bonferroni’s multiple comparison ad hoc post-test or unpaired Student’s t test, as appropriate. Statistically significant differences between data sets were assumed at p<0.05; (n) corresponds to the number of independent measurements.

## Results

### LIPT2 is expressed in the mitochondrion

To determine the subcellular localization of LIPT2, mitochondrial, cytosolic and microsomal fractions were prepared from total cellular protein extracts of native (untransfected) HEK 293 Phoenix cells and assayed for LIPT2 expression by western blot. Detectable levels of LIPT2 were found only in the complex IV-enriched mitochondrial fraction (Fig 2A).

The antibody used in western blot analyses failed to detect endogenous LIPT2 expression by immunocytochemistry (data not shown). Therefore, to confirm the subcellular localization of LIPT2 assessed by western blot and identify the domains determining the targeting to the mitochondrion, plasmids encoding the transfection marker EYFP fused either to the N or the C-terminus of LIPT2 (EYFP-LIP2 and LIPT2-EYFP, respectively) were generated and transfected in HeLa cells. The co-localization of EYFP-LIPT2, LIPT2-EYFP and the sole transfection marker EYFP with the mitochondria was determined 24, 48 and 72 hours after transfection (Fig 2B–2E). EYFP is a cytosolic protein and does not preferentially co-localize with the mitochondrion. The co-localization parameters (see Materials and methods) evidenced that only LIPT2-EYFP reached the mitochondria. While not being obvious 24 hours after transfection (Fig 2C), the mitochondrial targeting of LIPT2-EYFP was first detected 48 hours (Fig 2D) and was more evident 72 hours after transfection (Fig 2E). Similar results were obtained with HEK 293 Phoenix cells (S1 Fig). These findings confirm that LIPT2 targets the mitochondrion. EYFP-LIPT2 did not reach the mitochondrial compartment (Fig 2C–2E), suggesting that a free N-terminus is an essential requirement for LIPT2 mitochondrial targeting.

### LIPT2 amino acids 1–31 represent a mitochondrial targeting sequence

**Fig 3:**
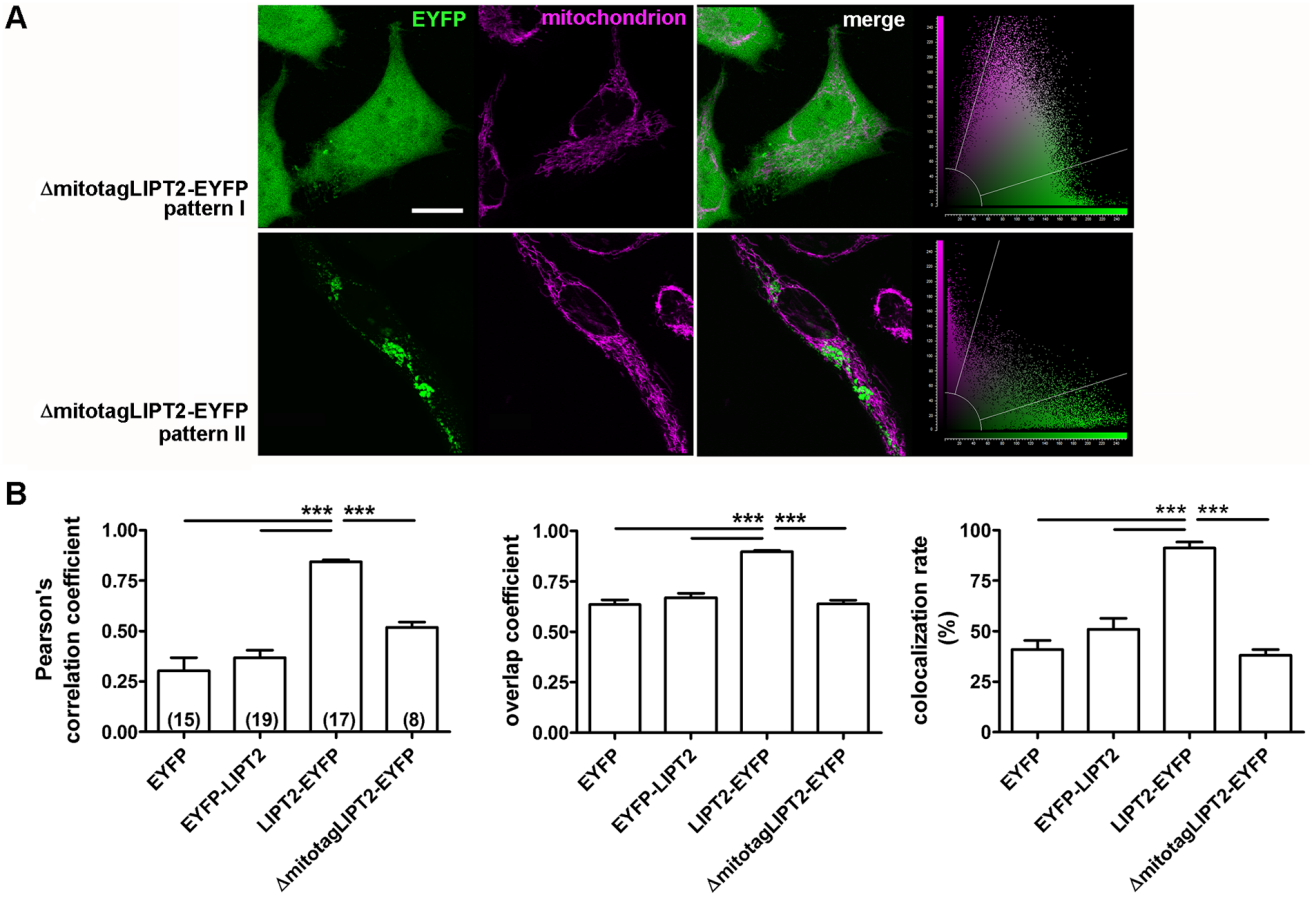
Removal of amino acids 1–31 prevents the targeting of LIPT2 to the mitochondrion. (A) From left to right: fluorescent signal of EYFP (green) and the mitochondrial marker (magenta) in HeLa cells expressing LIPT2-EYFP void of the amino acids 1–31 (ΔmitotagLIPT2-EYFP) for 72 hours, corresponding merge image and scatter plot. Scale bar: 20 μm. (B) Pearson’s correlation coefficient, overlap coefficient and co-localization rate (%) referred to the co-localization of ΔmitotagLIPT2-EYFP and the mitochondrion determined in HeLa cells 72 hours after transfection. (n) indicates the number of cells. ***: p<0.001, one-way ANOVA with Bonferroni’s post-test.

In HeLa cells, expression of the LIPT2-EYFP construct in which amino acids 1–31 were removed (ΔmitotagLIPT2-EYFP, see Materials and methods) led to either a diffuse distribution pattern not different from that shown by EYFP (pattern I, Fig 3A) or the formation of intracellular vesicular bodies, most likely originating from the endoplasmic reticulum (pattern II, Fig 3A). In both cases, colocalization with the mitochondria was prevented (Fig 3B), thus evidencing that amino acids 1–31 are necessary for the mitochondrial targeting of LIPT2.

**Fig 4:**
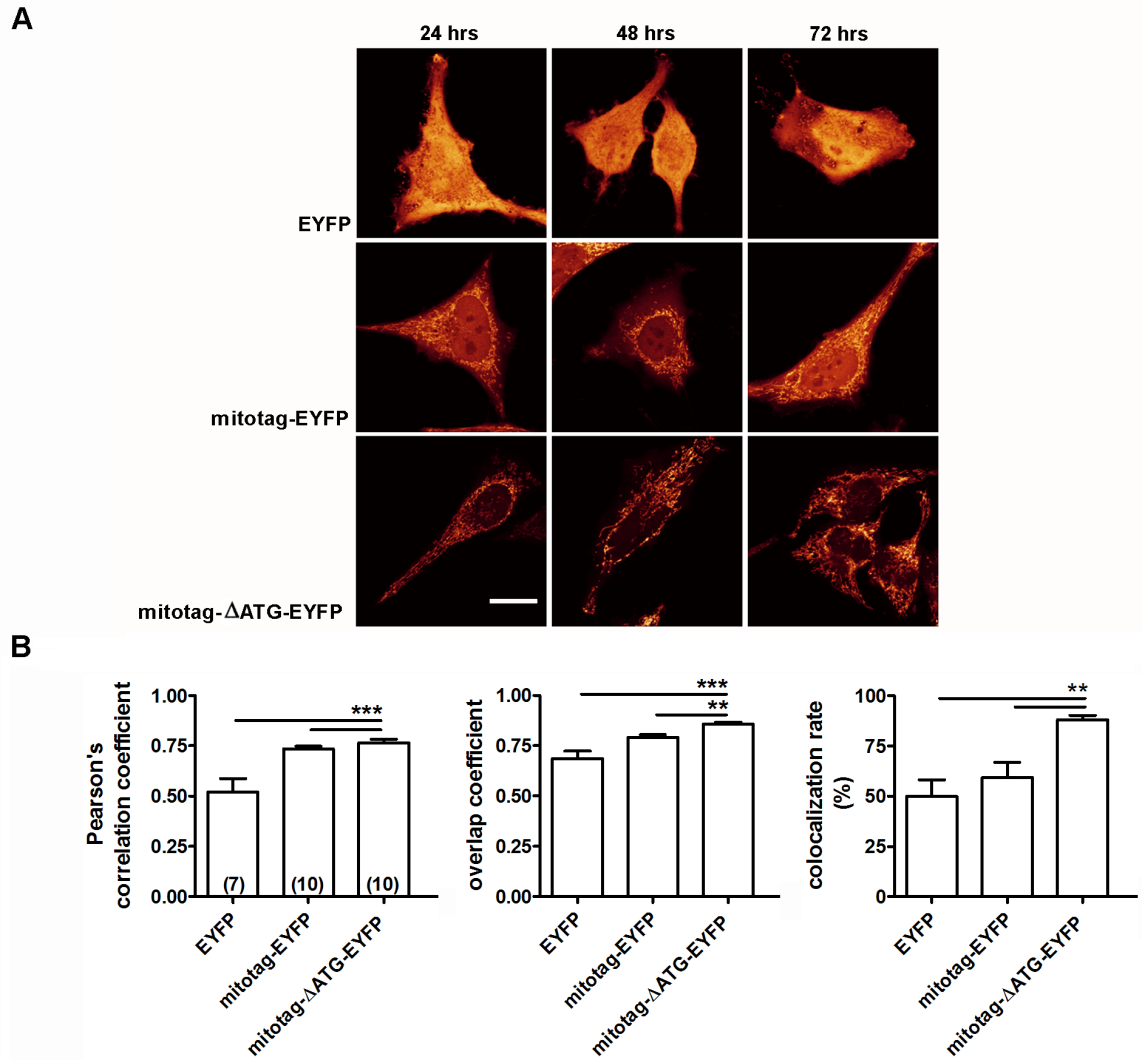
Amino acids 1–31 of LIPT2 target EYFP to the mitochondrion. (A) Fluorescent signal of EYFP, mitotag-EYFP, mitotag-ΔATG-EYFP after 24 (left), 48 (middle) and 72 hours (right) transfection in HeLa cells. Scale bar: 20 μm. (B) Pearson’s correlation coefficient, overlap coefficient and co-localization rate (%) referred to the co-localization of EYFP, mitotag-EYFP, mitotag-ΔATG-EYFP and the mitochondrion determined in HeLa cells 72 hours after transfection. (n) indicates the number of cells. **: p<0.01, ***: p<0.001, one-way ANOVA with Bonferroni’s post-test.

To confirm these findings, a construct was generated in which LIPT2 amino acids 1–31 were fused to the N-terminus of EYFP (mitotag-EYFP, see Materials and methods). Expression of the mitotag-EYFP construct in HeLa cells for 24–72 hours led to a clear fluorescent labeling of mitochondria. The extra-mitochondrial signal was reduced by removal of the initiation of translation (START) codon (ATG) of EYFP (Fig 4). These results show that LIPT2 amino acids 1–31 are sufficient for EYFP mitochondrial targeting but that a detectable amount of untagged EYFP can be produced if a START codon is located immediately downstream of the mitochondrial targeting sequence. Similar findings were obtained using the unrelated fluorescent protein dsRed instead of EYFP (data not shown), thus evidencing that LIPT2 amino acids 1–31 function as a mitochondrial targeting sequence independently of the protein to which they are fused.

### LIPT2 mitochondrial mis-targeting activates the apoptotic volume decrease (AVD) current

**Fig 5:**
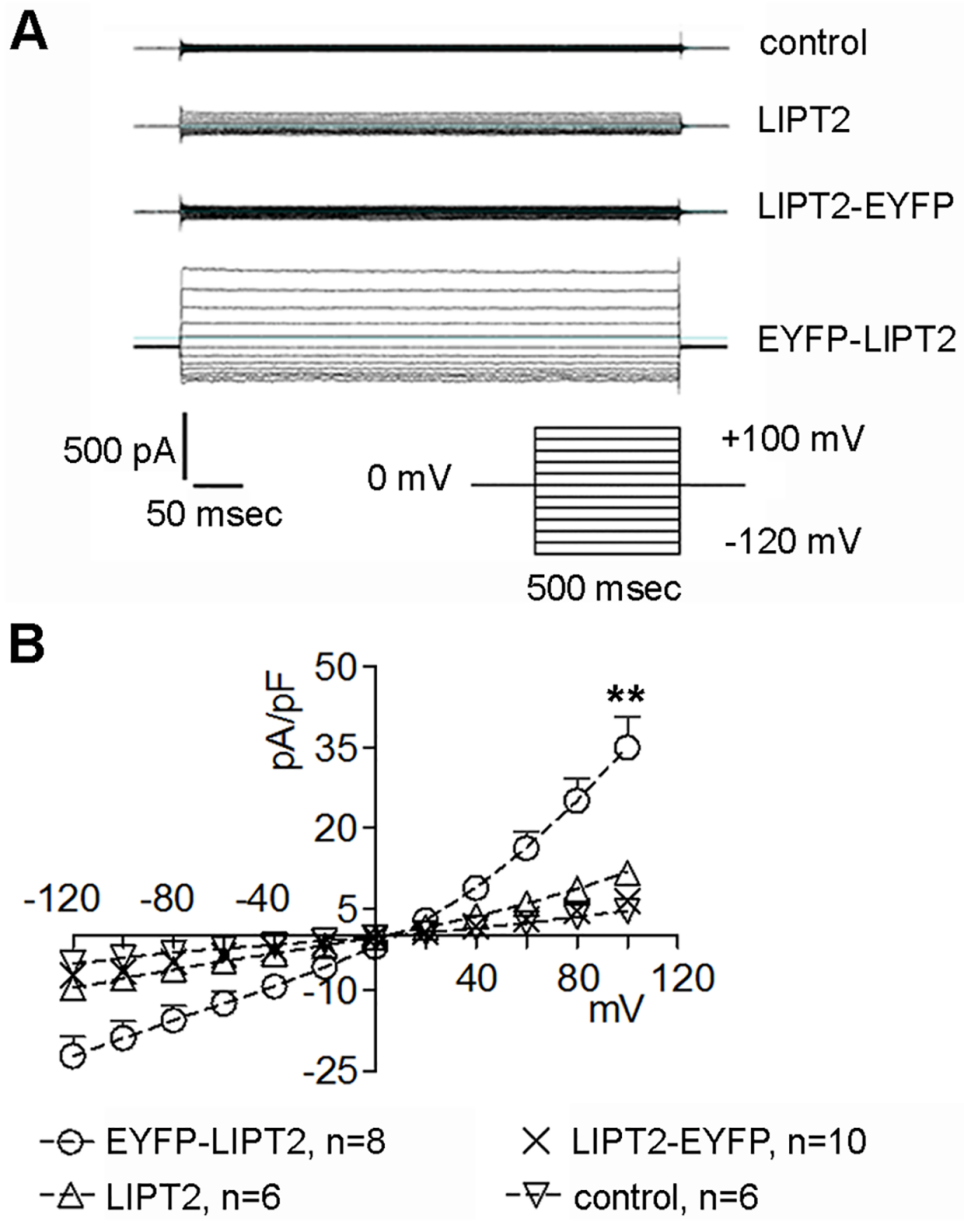
Apoptotic volume decrease (AVD) current is stimulated in EYFP-LIPT2 expressing cells. (A) Original current recordings obtained in whole-cell configuration from HEK 293 Phoenix cells kept in isotonic bath solution and stimulated with the pulse protocol represented in the inset. Cells were transfected for 48 hours with a fluorescent transfection marker without (control) or with LIPT2 or the fusion proteins LIPT2-EYFP or EYFP-LIPT2, as indicated. (B) Current density (pA/pF) to voltage (mV) relationship of control, LIPT2, LIPT2-EYFP and EYFP-LIPT2 expressing cells. (n) indicates the number of cells. **: p<0.01 compared to control, LIPT2 and LIPT2-EYFP, unpaired Student’s t test.

The presence of mitochondrion-resident proteins within the cytosol may trigger both caspase-dependent and independent pathways leading to apoptotic cell death [27]. The activation of the Apoptotic Volume Decrease (AVD) current is an early event in apoptosis [28,29]. Therefore, we investigated if the presence of LIPT2 within the cytosol leads to an activation of the AVD current. HEK 293 Phoenix cells expressing the sole transfection marker (control) or the transfection marker and LIPT2, or the fusion proteins LIPT2-EYFP and EYFP-LIPT2 where voltage clamped with the whole-cell patch-clamp technique using intracellular (pipette filling) and extracellular (bath) solutions suitable for isolating chloride currents and in the absence of osmotic gradients between the intracellular and extracellular milieu (see Materials and methods). In these conditions, a current with the biophysical fingerprints of the AVD current (including outward rectification, slow time-dependent inactivation at potentials higher than +40 mV and reversal potential compatible with an ionic selectivity for Cl^-^) was detected in cells expressing EYFP-LIPT2, *i*.*e*. the construct in which the LIPT2 mitochondrial targeting sequence is masked by EYFP (Fig 5). AVD current activation was not seen in control or LIPT2-EYFP expressing cells. In LIPT2 expressing cells, the current magnitude, although significantly higher compared to that measured in control cells, was greatly reduced compared to that measured in EYFP-LIPT2 expressing cells (Fig 5). These findings indicate that prevention of LIPT2 mitochondrial targeting activates the AVD current, which is suppressed when LIPT2 is correctly localized to the mitochondrion.

### LIPT2 mitochondrial mis-targeting activates the swelling-activated chloride current IClswell

**Fig 6:**
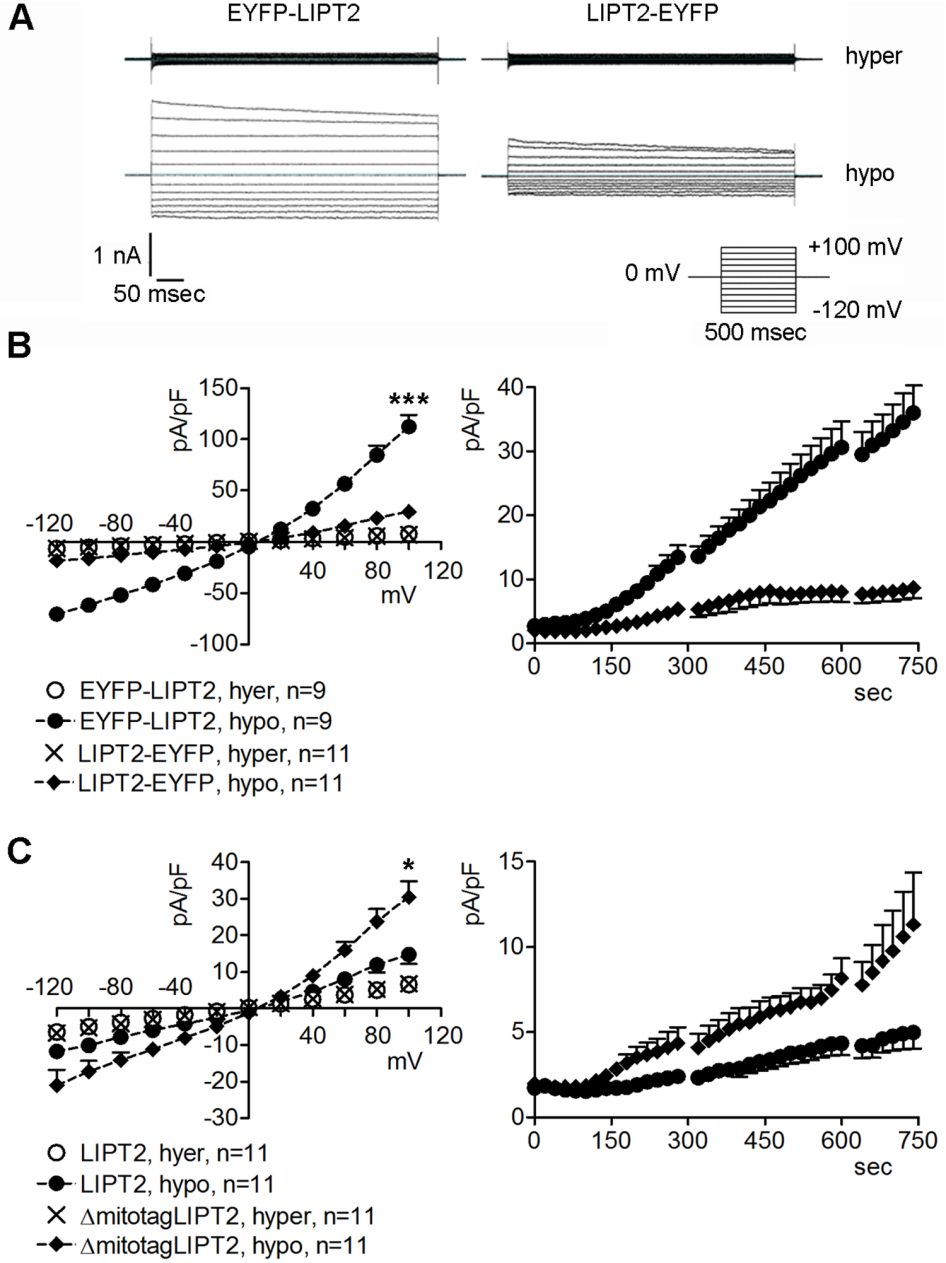
IClswell current is over-stimulated in EYFP-LIPT2 and ΔmitotagLIPT2 expressing cells. (A) Original current recordings obtained in whole-cell configuration from HEK 293 Phoenix cells in hypertonic (hyper) and hypotonic (hypo) bath solution following stimulation with the pulse protocol represented in the inset. Cells were transfected for 48 hours with EYFP-LIPT2 or LIPT2-EYFP, as indicated. (B) Current density (pA/pF) to voltage (mV) relationship measured in hypertonic solution and after a 10 minutes exposure to a hypotonic solution (left) and current density (pA/pF) to time (sec) relationship measured in hypotonic solution (right) in cells expressing EYFP-LIPT2 or LIPT2-EYFP. C, current density (pA/pF) to voltage (mV) relationship measured in hypertonic solution and after a 10 minutes exposure to a hypotonic solution (left) and current density (pA/pF) to time (sec) relationship measured in hypotonic solution (right) in cells expressing LIPT2 or ΔmitotagLIPT2. In B, left, ***: p<0.001 at all applied voltages except for 0 mV compared to LIPT2-EYFP, hypo, unpaired Student’s t test, and right, p<0.001 when time >160 sec, unpaired Student’s t test. In C, left, *: p<0.05 at all applied voltages except for 0 mV compared to LIPT2, hypo, unpaired Student’s t test, and right, p<0.05 when time >360 sec and <660 sec, unpaired Student’s t test. (n) indicates the number of cells.

The swelling-activated chloride current IClswell, which is stimulated after exposure of cells to an osmotic imbalance between the intracellular and the extracellular milieu, is an essential determinant of the ubiquitous homeostatic function of the Regulatory Volume Decrease (RVD) [30,31]. The AVD current and IClswell show the same biophysical properties and most likely rely on the same molecular entity [28,29,32]. Accordingly, induction of AVD current is coupled to a facilitation of RVD and prevented by known blockers of IClswell [33]. Apoptosis inducers rapidly activate IClswell [34]. Based on these considerations, and having observed that preventing LIPT2 mitochondrial trafficking activates the AVD current, we monitored the activation of IClswell in HEK 293 Phoenix cells expressing EYFP-LIPT2 or LIPT2-EYFP (Fig 6A and 6B). Cells were voltage clamped with the whole-cell patch-clamp technique and initially kept in hypertonic solution (see Materials and methods). In this condition, no obvious currents were detected (Fig 6A), according to previous observations [20,22,25]. Then, IClswell was activated following a 28% reduction (100 mOsm) of the extracellular osmolarity *via* omission of mannitol from the bath solution. This maneuver activated IClswell in both EYFP-LIPT2 and LIPT2-EYFP transfected cells, however, 10 minutes after stimulation of cells with hypotonic solution, the current magnitude was significantly higher and the activation kinetic was significantly faster in EYFP-LIPT2 expressing cells (Fig 6B). Similarly, in cells expressing LIPT2 void of the mitochondrial tag, the current magnitude was significantly higher and the activation kinetic was significantly faster compared to that measured in cells expressing the full-length LIPT2 (Fig 6C). These findings indicate that deranged mitochondrial targeting of LIPT2 facilitates the activation of IClswell.

### LIPT2 does not *per se* modify IClswell

**Fig 7:**
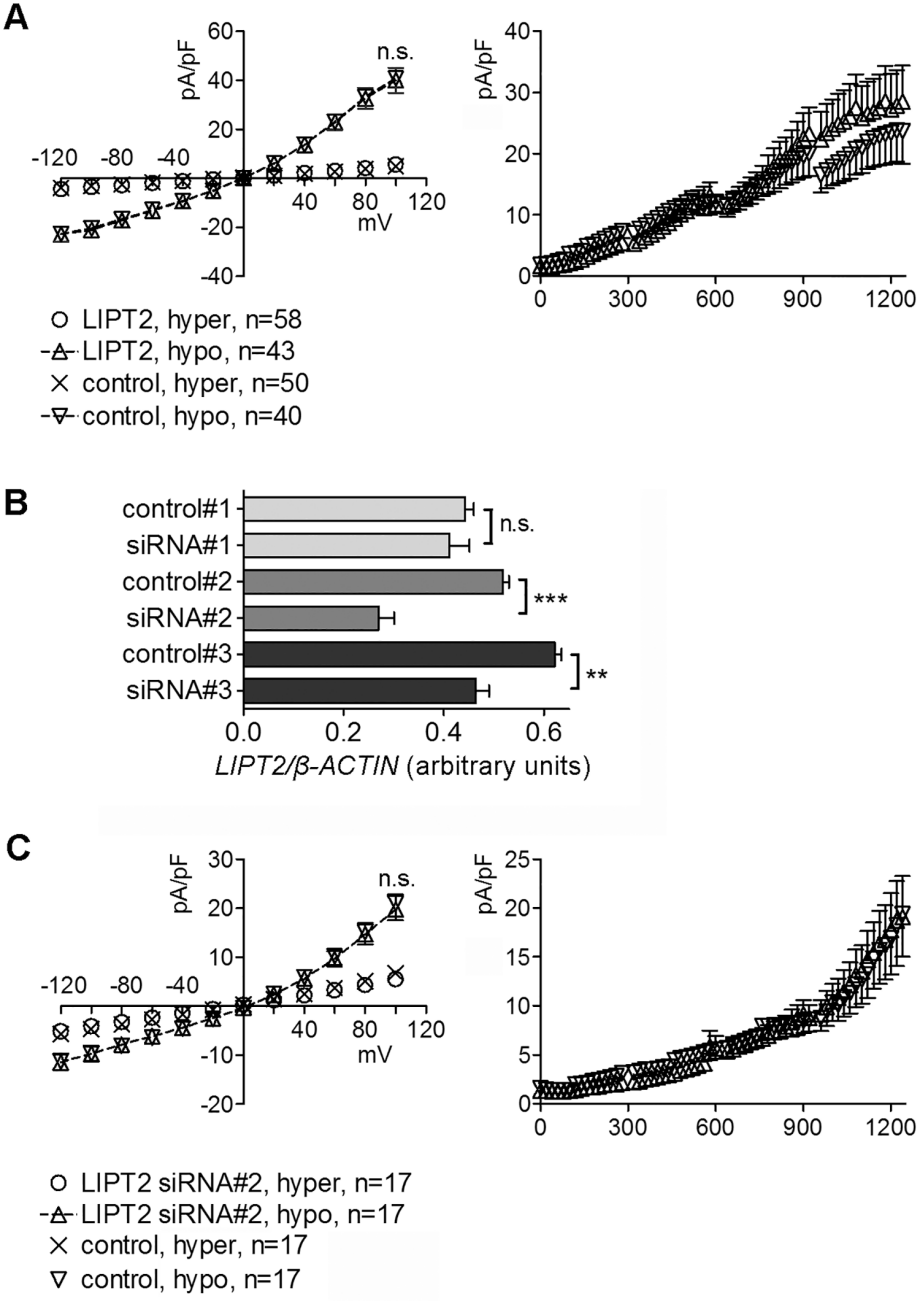
IClswell current is not affected following alteration of LIPT2 expression. (A) Current density (pA/pF) to voltage (mV) relationship measured in hypertonic solution and after a 10 minutes exposure to a hypotonic solution (left) and current density (pA/pF) to time (sec) relationship measured in hypotonic solution (right) in HEK 293 Phoenix cells transfected for 48 hours with LIPT2 or a transfection marker (control). (B) Levels of the transcript of *LIPT2* detected by RT-PCR in HEK 293 Phoenix cells transfected for 48 hours with siRNA #1, 2 and 3 or control siRNA (control) and normalized for the levels of the transcript of the housekeeping gene *β-ACTIN*. Gene silencing of 7, 48 and 25% was obtained, respectively. (C) Current density (pA/pF) to voltage (mV) relationship measured in hypertonic solution and after a 10 minutes exposure to a hypotonic solution (left) and current density (pA/pF) to time (sec) relationship measured in hypotonic solution (right) in cells transfected with siRNA #2 or a control siRNA (control). n.s., not significant, **, ***: p<0.01, p<0.001, unpaired Student’s t test. (n) in A and C indicates the number of cells. In B, n = 3 for each condition.

To establish if the results shown in Fig 6 could be explained by a suppression of IClswell by LIPT2 forms correctly targeting to the mitochondrion (*i*.*e*. LIPT2 and LIPT2-EYFP) instead of an activation of IClswell by the LIPT2 forms of which the mitochondrial targeting is prevented (*i*.*e*. ΔmitotagLIPT2 and EYFP-LIPT2), we verified if LIPT2 may *per se* modify the biophysical properties of IClswell. Altered LIPT2 expression levels were obtained following transfection of LIPT2 (Fig 7A) or siRNAs (Fig 7B and 7C). LIPT2 overexpression (Fig 7A) or 48% gene expression silencing with siRNA #2 (Fig 7C) did not modify the magnitude or the activation kinetics of IClswell.

### Preventing LIPT2 mitochondrial targeting induces early and late apoptotic changes

**Fig 8:**
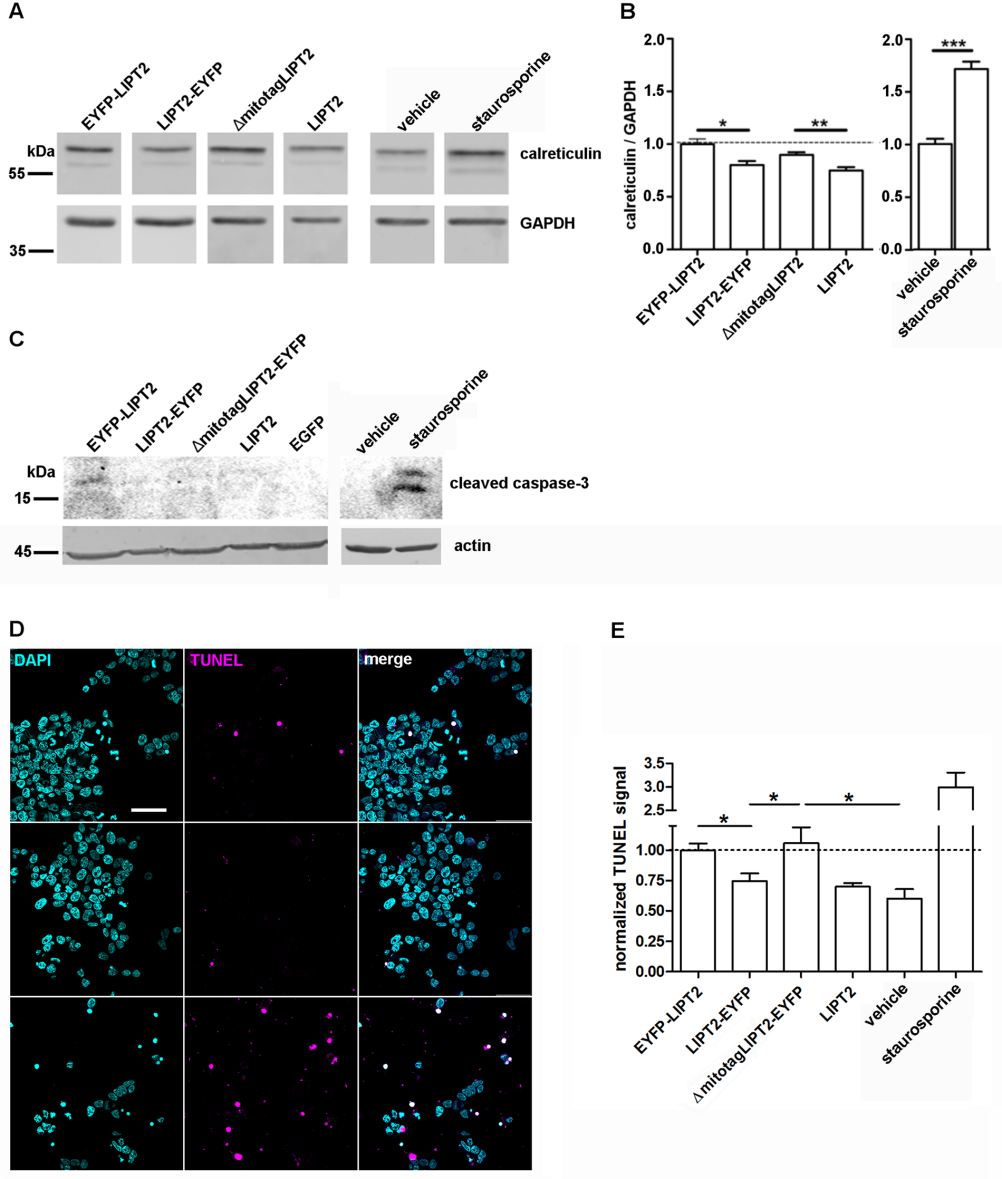
Apoptosis is induced in EYFP-LIPT2 and ΔmitotagLIPT2 expressing cells. (A) Representative immunodetection of calreticulin in whole cell lysates from HEK 293 Phoenix cells 48 hours after transfection with the indicated constructs or from cells treated with the vehicle or 20 μM staurosporine for 4 hours. The lower panels correspond to the signal of the housekeeping protein GAPDH. (B) The expression levels of calreticulin were quantified by densitometry and normalized to GAPDH. n = 3, *: p<0.05, **: p<0.01, ***: p<0.001, two-tailed, unpaired Student’s t test. (C) Immunodetection of cleaved caspase-3 and the housekeeping protein β-actin in whole cell lysates from HEK 293 Phoenix cells 48 hours after transfection with the indicated constructs or from cells treated with the vehicle or 20 μM staurosporine for 4 hours. The image is representative of three independent experiments. (D) From left to right: fluorescent signal of DAPI (cyan), TUNEL (magenta) and corresponding merge image of HEK 293 Phoenix cells expressing EYFP-LIPT2 (top), LIPT2-EYFP (middle), and ΔmitotagLIPT2 (bottom) for 48 hours. Scale bar: 50 μm. (E) Normalized TUNEL signal intensity of cells transfected with the indicated constructs or treated overnight with the vehicle or 2 μM staurosporine. 12≤n≤25 indicates the number of imaging fields from 3 independent experiments. *: p<0.05, one-way ANOVA with Bonferroni’s post-test.

Increased expression, cell surface exposure and release of calreticulin are early markers of apoptosis [35]. Accordingly, an increased expression of calreticulin could be detected in whole cell lysates from cells stimulated with staurosporine (Fig 8A and 8B). Calreticulin expression levels were quantified by western blot and were found to be significantly elevated in cells expressing EYFP-LIPT2 and ΔmitotagLIPT2 (Fig 8A and 8B). In addition, cleaved caspase-3 could be detected in EYFP-LIPT2 expressing cells (Fig 8C).

DNA fragmentation, the ultimate step of apoptotic signaling cascades, was detected by the TUNEL assay in EYFP-LIPT2, LIPT2-EYFP, ΔmitotagLIPT2 and LIPT2 expressing cells. The TUNEL signal was significantly elevated in EYFP-LIPT2 and ΔmitotagLIPT2 transfected cells and was associated with either small and condensed nuclei or the presence of apoptotic bodies (Fig 8D and 8E). These findings provide evidence that apoptosis is induced in cells in which the targeting of LIPT2 to the mitochondrion is prevented.

### Preventing LIPT2 mitochondrial targeting causes mitochondrial membrane potential collapse

**Fig 9:**
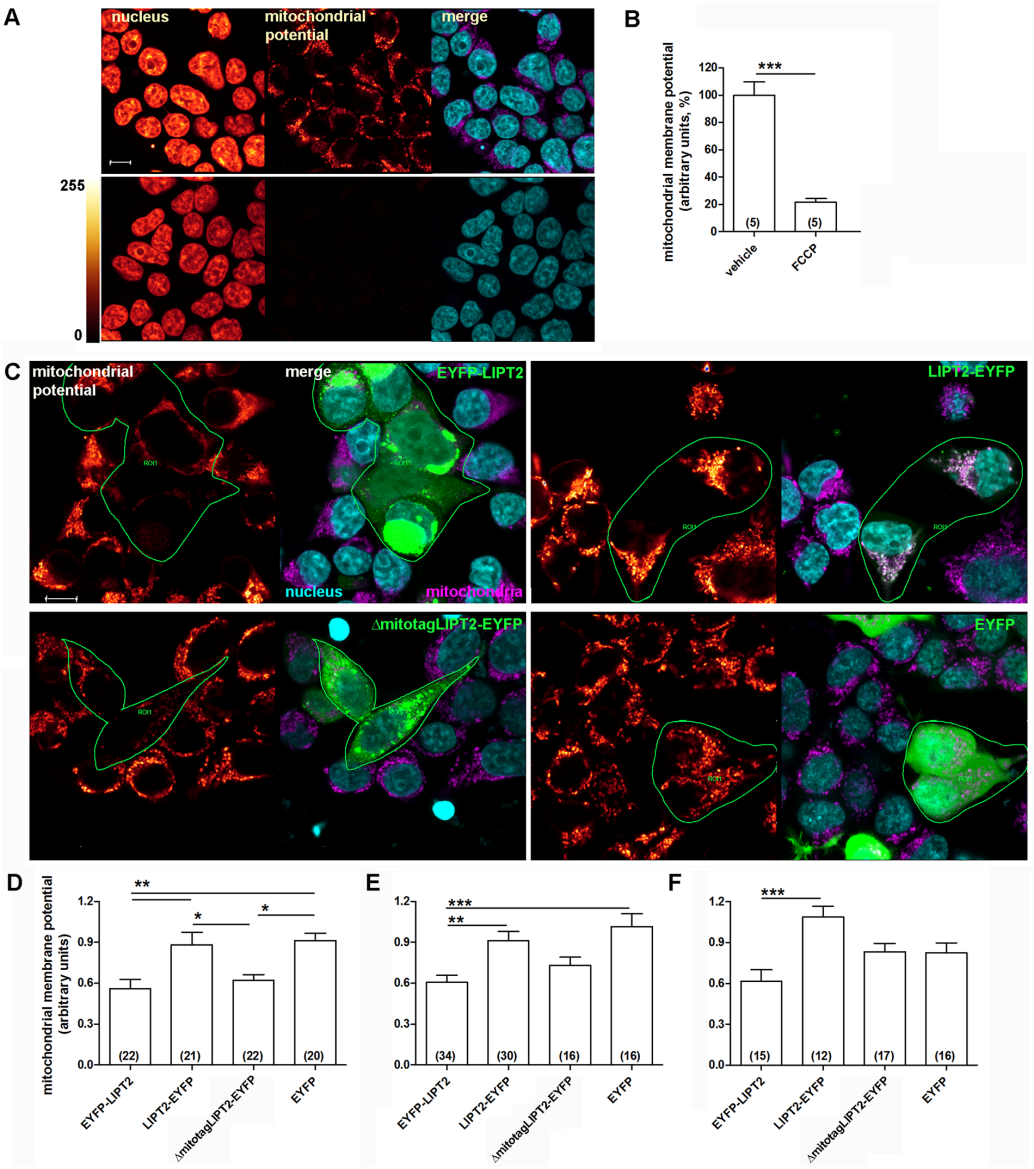
Mitochondrial membrane potential depolarization is observed in EYFP-LIPT2 and ΔmitotagLIPT2 expressing cells. (A) From left to right: fluorescence intensity of Hoechst (nucleus), Mito Tracker Deep Red (mitochondrial potential) and corresponding merge image (nucleus in cyan and mitochondria in magenta) of HEK 293 Phoenix cells treated with the vehicle (top panels) or the uncoupling agent FCCP (bottom panels) for 30 minutes. Scale bar: 10 μm. (B) Mitochondrial membrane potential in vehicle and FCCP-treated cells. (n) refers to the number of fields imaged. ***: p<0.001, two-tailed, unpaired, Student’s t test. (C) Fluorescence intensity of Mito Tracker Deep Red (mitochondrial potential) in cells expressing the indicated constructs for 48 hours and the corresponding merge image, with transfected cells (green) within a ROI, mitochondria in magenta and nuclei counterstained with Hoechst (cyan). Scale bar: 10 μm. (D-F) Mitochondrial membrane potential determined 24, 48 and 72 hours after transfection, respectively. (n) indicates the number of cells. *: p<0.05, **: p<0.01, ***: p<0.001, one-way ANOVA with Bonferroni’s post-test.

Staining of mitochondria with Mito Tracker Deep Red requires an intact mitochondrial membrane potential and fluorescence intensity variations of this dye can be used to monitor mitochondrial membrane potential changes. Accordingly, in cells treated with the uncoupling agent FCCP, the fluorescence intensity of Mito Tracker Deep Red was dramatically reduced (Fig 9A and 9B). Significant reductions in the mitochondrial membrane potential were also detected in cells expressing EYFP-LIPT2 and ΔmitotagLIPT2 (Fig 9C–9F).

## Discussion

Mitochondria are evolutionary ancient organelles involved in multiple crucial functions of the eukaryotic cell, including energy production, synthesis of lipids and other biomolecules and orchestration of the apoptotic process. The human mitochondrial genome encodes only 13 of the over 1,000 mitochondrial proteins. Therefore, the large majority of the mitochondrial proteome is represented by nuclear encoded proteins translated on cytosolic ribosomes and actively imported into mitochondrial sub-compartments [36]. The classical type of mitochondrial targeting signal is represented by an N-terminally located presequence of 15–50 amino acids that forms positively charged amphipathic α helices. Sequence, net charge and structure of the presequence are critical for the interaction with the translocator of the outer and inner membrane (TOM and TIM) macromolecular complexes in a sequential manner. Once the preprotein reaches the mitochondrial matrix, the presequence is often proteolytically removed by mitochondrial processing peptidases (MPP). As many as 40% of all mitochondrial proteins do not possess N-terminal extensions, but include internal non-cleavable targeting signals which are variable in type and number [37;38]. LIPT2 is a nuclear encoded protein of which the mitochondrion is the main location (Fig 2) and LIPT2 amino acids 1–31 represent its mitochondrial targeting presequence. Removal of amino acid 1–31 is sufficient to abrogate the mitochondrial targeting of LIPT2 (Fig 3), therefore evidencing that the function of the targeting presequence cannot be fulfilled by internal signals. This concept is further supported by the evidence that the LIPT2 presequence can drive unrelated cytoplasmic proteins to the mitochondrion (Fig 4). LIPT2 amino acids 1–31 are thus necessary and sufficient for protein targeting to the mitochondrion.

The first 18 amino acids of the precursor to subunit IV of Cytochrome oxidase represent a classical signal sequence for protein import into the mitochondrion. This sequence can form an amphipathic α helix with positively charged residues (Arg 5, Arg 9, Lys 12 and Arg 16) on one side of the helix and non-polar residues on the other side [39]. Interestingly, Arg residues are over-represented among the first 31 N-terminal amino acids of LIPT2. When this segment is organized into an α helix, those amino acid residues that would bear a positive charge at physiological pH (Arg 2, 7, 10, 13, 25, 28 and 29; pKa of the amino group of the side chain of Arg is 12.48) appear to be clustered on one face of the helix, with the non-polar residues (Met 1, Pro 4, Ala 5, Leu 8, Val 9, Leu 11, Gly 12, Pro 15, Leu 19, Leu 20, Leu 22, Trp 26, Leu 27 and Leu 30) on the opposite face (S2 Fig), therefore nicely mirroring the characteristics of a classical signal sequence for protein import into the mitochondrial matrix.

Given the mitochondrial localization of LIPT2, a direct molecular interaction with B-myc, which is a nuclear protein, is surprising [15]. Whether this interaction truly occurs in native cell systems or is a false positive generated by displacement of LIPT2 and/or B-myc from their physiological compartments due to the generation of fusion proteins (false positives are not uncommon with two-hybrid based protein-protein interaction screenings [40]) remains to be established.

Staining of mitochondria is often required to investigate their morphology, number and localization. Fluorescent staining of mitochondria in living cells with cationic dyes, such as rhodamine 123 and other patented molecules, requires an intact mitochondrial membrane potential [41]. In addition, some of these dyes are not retained within the mitochondrion following cell fixation and/or permeabilization. Fusion constructs consisting of the mitochondrial targeting sequence of LIPT2 and the fluorescent proteins EYFP and DsRed were easily expressed in living cells (Fig 4). Staining of the mitochondrial compartment by these fluorescent probes does not depend on the mitochondrial membrane potential and is therefore suitable for use in conditions where the mitochondrial membrane potential is altered, for example, following pharmacological treatment or in cell models of pathological conditions where mitochondrial function is compromised. Besides providing optimal signal-to-noise ratio, this strategy allows for fluorophore retention within the mitochondrial compartment after cell fixation and/or permeabilization and is therefore compatible with conventional sample processing for immunocytochemistry and other techniques and could in principle be used in combination with other established staining methods.

Mitochondria control cell fate by inducing the apoptotic cascade following mitochondrial outer membrane permeabilization (MOMP). Mitochondria sequester holocytochrome c, an essential electron shuttle of the respiratory chain, in the mitochondrial intermembrane space. Following MOMP, holocytochrome c and other mitochondrial proteins are released into the cytosol, where they are thought to promote the activation of caspase-dependent and independent cell death pathways. The caspase activation cascade leads to the activation of caspase-3, a central executioner of apoptosis, partially or totally responsible for the proteolytic cleavage of several proteins in the late stages of the apoptotic process. Oligonucleosomal DNA fragmentation is the ultimate step on which caspase-dependent and independent cell death pathways culminate [42–44]. Based on this knowledge, we verified if the presence of the mitochondrial protein LIPT2 in extra-mitochondrial compartments triggers apoptosis. As already mentioned, cell shrinkage or apoptotic volume decrease (AVD) is one of the obligate initial events and most reproducible signs in the apoptotic process and is dependent upon normotonic activation of a chloride conductance resembling the hypotonicity-activated chloride current IClswell [28,29,32,33,45–47]. Activation of AVD current was detected in cells expressing a LIPT2 construct incapable of mitochondrial targeting (EYFP-LIPT2, Fig 5). Conversely, LIPT2 constructs of which the mitochondrial targeting was not affected (LIPT2 and LIPT2-EYFP, Fig 5) did not activate the AVD current. These evidences point to the fact that LIPT2 does not *per se* activate AVD, but AVD is triggered when LIPT2 fails to reach the mitochondria. This indicates that the presence of LIPT2 in an extra-mitochondrial compartment is capable of triggering apoptosis.

The AVD and IClswell currents most likely rely on the same molecular entities. As RVD is dependent upon IClswell activation, induction of AVD is coupled to a facilitation of RVD [33;34]. LIPT2 constructs incapable of mitochondrial targeting (EYFP-LIPT2 and ΔmitotagLIPT2, Fig 6) led to an over-activation of IClswell current compared to what was observed with LIPT2 constructs with normal mitochondrial targeting (LIPT2 and LIPT2-EYFP, Fig 6). Being that LIPT2 *per se* is unable of modulating IClswell magnitude and activation kinetics (Fig 7), these data indicate that IClswell is facilitated when LIPT2 does not reach the mitochondria and further support a role of LIPT2 in triggering AVD. Accordingly, increased expression of calreticulin and DNA fragmentation, hallmarks of the early and end stages of apoptosis respectively, were observed in cells expressing LIPT2 constructs incapable of mitochondrial targeting (EYFP-LIPT2 and ΔmitotagLIPT2, Fig 8), therefore supporting the concept that apoptosis is triggered when LIPT2 fails to reach the mitochondria.

Mutations in *LIPT1* and *LIPT2* genes begin to emerge as causes of infant death or severe disease [11,12,48,49] and have been linked to loss of function of the corresponding proteins. One of the two mutations in *LIPT2* (c.T89C) described by Habarou et al. is predicted to lead to an amino acid substitution within the mitochondrial targeting sequence of the protein (p.Leu30Pro) and may therefore affect its trafficking to the mitochondrion. We described here that the presence of LIPT2 outside the mitochondrial compartment may lead to apoptotic cell death. Apoptosis is consistent with and may contribute to the pathological phenotype described in the context of dysfunction of the lipoic acid biosynthesis pathways.

As mentioned earlier, caspase-3 is an end-stage effector of mitochondrion, receptor and ER stress-mediated apoptotic pathways and is activated by proteolytic cleavage [43]. We therefore verified if caspase-3 activation was observed in HEK 293 Phoenix cells expressing apoptosis-inducing LIPT2 constructs. Cleaved caspase-3 was detected in whole cell lysates derived from cells treated for 4 hours with 20 μM staurosporine, an established inducer of apoptosis, and cells expressing EYFP-LIPT2 (Fig 8C), further supporting the activation of a caspase-dependent apoptotic pathway following impeded LIPT2 mitochondrial trafficking.

Whole-field measurements of mitochondrial transmembrane potential (S1 File) did not reveal significant differences following expression of EYFP-LIPT2 or LIPT2 silencing (S3 Fig). In line, oxygen consumption (S1 File) was not significantly altered following expression of pro-apoptotic constructs or LIPT2 silencing (S4 Fig). However, in case of LIPT2 silencing, mitochondrial function could have been preserved by residual LIPT2. Also, possible alterations in mitochondrial function by pro-apoptotic constructs could have been masked by a transfection efficiency lower than 100%. Indeed, determination of mitochondrial membrane potential on a single-cell level evidenced mitochondrial membrane potential collapse specifically in cells expressing apoptosis-inducing LIPT2 constructs (EYFP-LIPT2 and ΔmitotagLIPT2-EYFP, Fig 9). These observations indicate that the mechanism by which the presence of LIPT2 outside the mitochondrion induces apoptotic cell death is most likely related to mitochondrial dysfunction.

Apoptosis can be induced by ER stress [43]. Therefore, we verified if caspase-12 cleavage, a hallmark of ER-stress [50], could preferentially be detected in cells expressing apoptosis-inducing LIPT2 constructs (EYFP-LIPT2 and ΔmitotagLIPT2-EYFP). Cleavage of caspase-12 was detected in all transfected cells, and not in native (untransfected) cells, thus indicating that ER stress is more liked to the transfection *per se*, rather to the expression of specific constructs (S5 Fig). As mentioned above, various pro-apoptotic stimuli are capable of inducing the release of specific proteins, including holocytochrome c, from the mitochondrion, and the presence of mitochondrial proteins within the cytosolic compartment can unequivocally be considered as a marker of mitochondrial damage and initiation of apoptotic cell death [43,51–53]. Therefore, we investigated if LIPT2 can be retrieved within the cytosol following an apoptotic stimulus. HEK 293 Phoenix cells were treated with 20 μM staurosporine or the vehicle, and the presence of LIPT2 and holocytochrome c in the cytosolic and mitochondrial fractions was assessed by western blot. As expected, holocytochrome c was retrieved only in the mitochondrial fraction of vehicle-treated cells and both in the cytosolic and mitochondrial fractions of staurosporine-treated cells (S5 Fig). However, LIPT2 was only retrieved in the mitochondrial fraction, and not in the cytosolic fraction, of staurosporine or vehicle-treated cells (S5 Fig). These findings led us to conclude that LIPT2 is not released into the cytosol following an apoptotic stimulus. This is consistent with a localization of LIPT2 within the mitochondrial matrix, differently from holocytochrome c, which is retrieved in the intermembrane space of non-apoptotic cells.

## Conclusions

The findings presented here show that LIPT2 is a protein expressed in the mitochondria of human cells. Amino acid 1–31 of LIPT2 represent the mitochondrial targeting sequence, and have been used to produce plasmid constructs that could be easily transfected in cells to obtain fluorescent labeling of mitochondria independent from the mitochondrial transmembrane potential.

Prevention of transit of LIPT2 to the mitochondrion results in apoptotic cell death, as revealed by activation of the AVD current, mitochondrial membrane potential collapse, caspase-3 cleavage and nuclear DNA fragmentation. Activation of AVD current in normotonic conditions was associated with over-activation of the chloride current IClswell elicited by a hypotonic challenge. We therefore suggest that over-activation of IClswell could be a useful tool to identify early stages of the apoptotic process.

Alteration of the LIPT2 mitochondrial targeting sequence should be considered in evaluating the impact of mutations leading to lipoic acid biosynthesis defects in humans.

## Supporting information

S1 Fig LIPT2-EYFP targets the mitochondrion. Pearson’s correlation coefficient, overlap coefficient and co-localization rate (%) referred to the co-localization of EYFP, EYFP-LIPT2 or LIPT2-EYFP and the mitochondrion determined in HEK 293 Phoenix cells (A), 24, (B), 48 and (C), 72 hours after transfection. (n) indicates the number of cells. *: p<0.05, **: p<0.01, ***: p<0.001, one-way ANOVA with Bonferroni’s post-test.
(TIF)

S2 Fig The mitochondrial targeting sequence of LIPT2 can generate an amphipathic α helix. (A) Amino acid sequence of LIPT2. The mitochondrial targeting sequence (amino acids 1–31) is highlighted in yellow. (B) Helical wheel projection of the mitochondrial targeting sequence of LIPT2, with the first (M1) and the last (Q31) amino acids in grey circles. In (A) and (B), the amino acid residues that would bear a positive charge at physiological pH (Arg 2, 7, 10, 13, 25, 28 and 298) are indicated in red, and the non-polar residues (Met 1, Pro 4, Ala 5, Leu 8, Val 9, Leu 11, Gly 12, Pro 15, Leu 19, Leu 20, Leu 22, Trp 26, Leu 27 and Leu 30) are indicated in blue. The helical wheel projection was generated according to Gene Runner, 3.05 and Helical Wheel Projections, Version: Id: wheel.pl,v 1.4 2009-10-20 21:23:36 don Exp, http://rzlab.ucr.edu/scripts/wheel/wheel.cgi.
(TIF)

S3 Fig Whole-field determination of mitochondrial membrane potential. (A) From left to right: fluorescent signal of Hoechst (nucleus), EYFP, Mito Tracker Deep Red (mitochondrial potential) and corresponding merge image of HEK 293 Phoenix cells transfected for 48 hours with the indicated constructs. Scale bar: 30 μm. (B) From left to right: fluorescent signal of Hoechst (nucleus), Mito Tracker Deep Red (mitochondrial potential) and corresponding merge image of HEK 293 Phoenix cells transfected for 48 hours with control siRNA and LIPT2 siRNA#2. (C) Mitochondrial membrane potential normalized for the cell density. No statistically significant differences between groups were found, one-way ANOVA with Bonferroni’s post-test, n = 8. (n) indicates the number of imaging fields.
(TIF)

S4 Fig Oxygen consumption in intact HEK 293 Phoenix cells determined by O2k high-resolution respirometry. (A) Representative traces of the respiration in intact cells. The blue line represents the oxygen concentration, the red line the oxygen flux. A coupling control protocol was applied after adding the cells into the O2k-chambers. (B) ROUTINE respiration (*R*) reflecting cellular oxygen consumption under near-physiological conditions. In this state, cells experience neither saturating substrate conditions nor are they challenged by elevated cellular ATP demand. (C) Free ROUTINE activity, calculated as the difference between *R* and LEAK (*L*) respiration (*R*-*L*). The latter represents the component of respiration not related to ATP production, but supporting proton transfer compensating for dissipative proton fluxes across the inner mitochondrial membrane. (D) Respiratory excess capacity, representing the difference between electron transfer system (ETS) capacity and *R*. ETS reflects maximum respiratory activity observed when the limitation imposed by the oxidative phosphorylation system is removed by uncoupling respiration from oxidative phosphorylation. All data for oxygen flow are corrected for residual oxygen consumption (obtained after the inhibition of the complexes I and III by rotenone and antimycin A, respectively) and for cell viability. Cells were transfected for 48 hours with the indicated constructs. Data are means ± SEM of n = 4 cell cultures per experimental group. No statistically significant differences between groups were found, one-way ANOVA.
(TIF)

S5 Fig LIPT2-induced apoptosis is not dependent on endoplasmic reticulum stress and LIPT2 is not released from the mitochondrion following induction of apoptosis. (A) HEK 293 Phoenix cells were transfected for 48 hours with the indicated constructs or left untransfected (native). Caspase-12 was immunodetected in whole cell lysates. Cleaved caspase-12 was retrieved in all samples from transfected cells, and not in native cells. The images are representative of 3 independent samples. (B) Untransfected HEK 293 Phoenix cells were treated either with 20 μM staurosporine or the vehicle for 4 hours. Cytochrome c could be detected in the mitochondrial fractions and in the cytosolic fraction of staurosporine-treated cells, but not in the cytosolic fraction of vehicle-treated cells. (C) In the same samples shown in (B), endogenous LIPT2 could only be detected in the mitochondrial fractions, but not in the cytosolic fractions of staurosporine or vehicle-treated cells. In (B) and (C), the housekeeping protein β-actin was immunodetected as a loading control.
(TIF)

S1 File Whole-field determination of mitochondrial membrane potential and measurements of mitochondrial function (oxygen consumption) in intact cells. (DOCX)
