# Supplementary figures and images for: Mis-targeting of the mitochondrial protein LIPT2 leads to apoptotic cell death
Source: PLoS One. 2017 Jun 19;12(6):e0179591. doi: 10.1371/journal.pone.0179591 (PMC5476274; doi:10.1371/journal.pone.0179591)

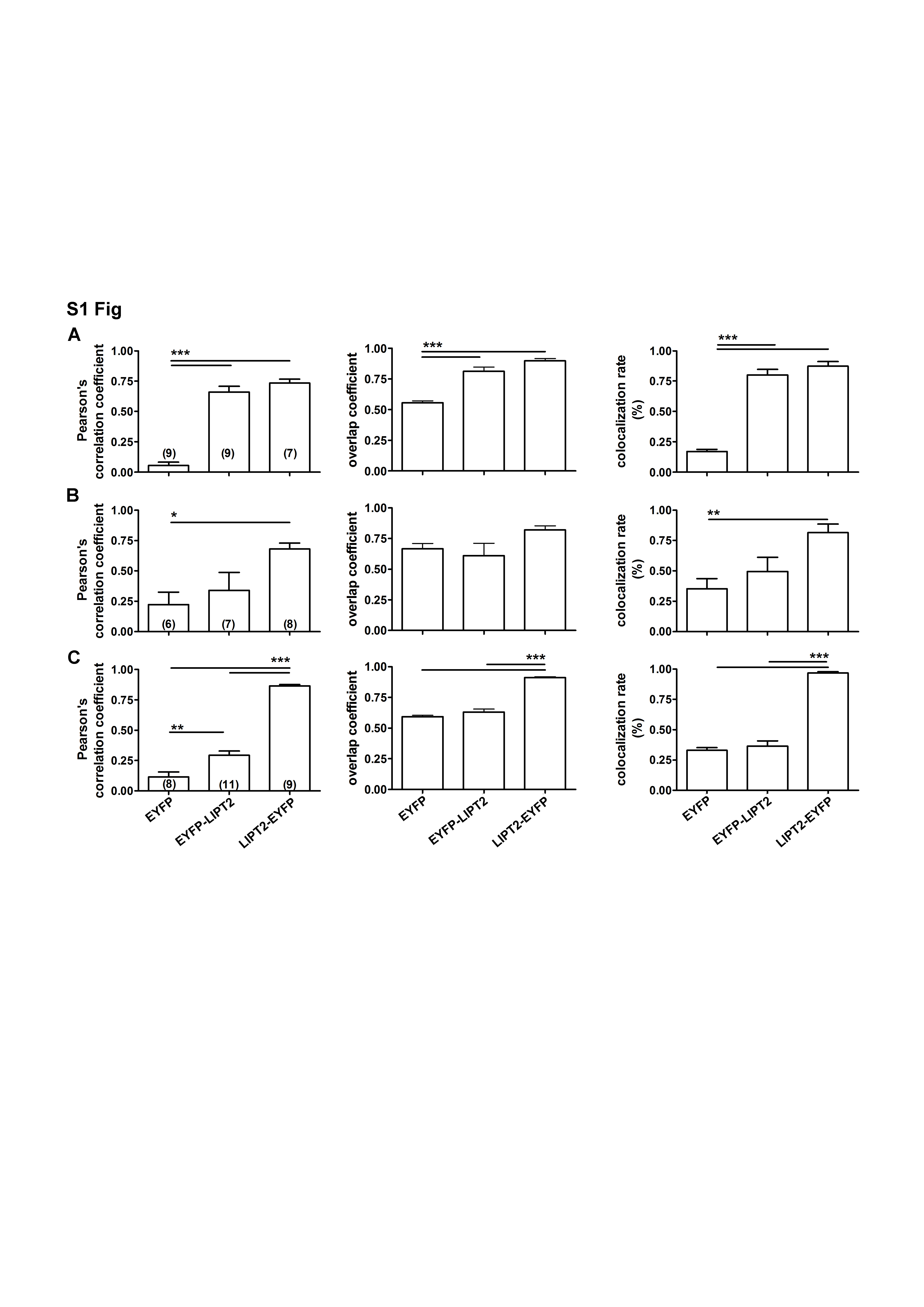

Supplement: S1 Fig — Pearson’s correlation coefficient, overlap coefficient and co-localization rate (%) referred to the co-localization of EYFP, EYFP-LIPT2 or LIPT2-EYFP and the mitochondrion determined in HEK 293 Phoenix cells (A), 24, (B), 48 and (C), 72 hours after transfection. (n) indicates the number of cells. *: p<0.05, **: p<0.01, ***: p<0.001, one-way ANOVA with Bonferroni’s post-test. (TIF) [file pone.0179591.s001.tif]

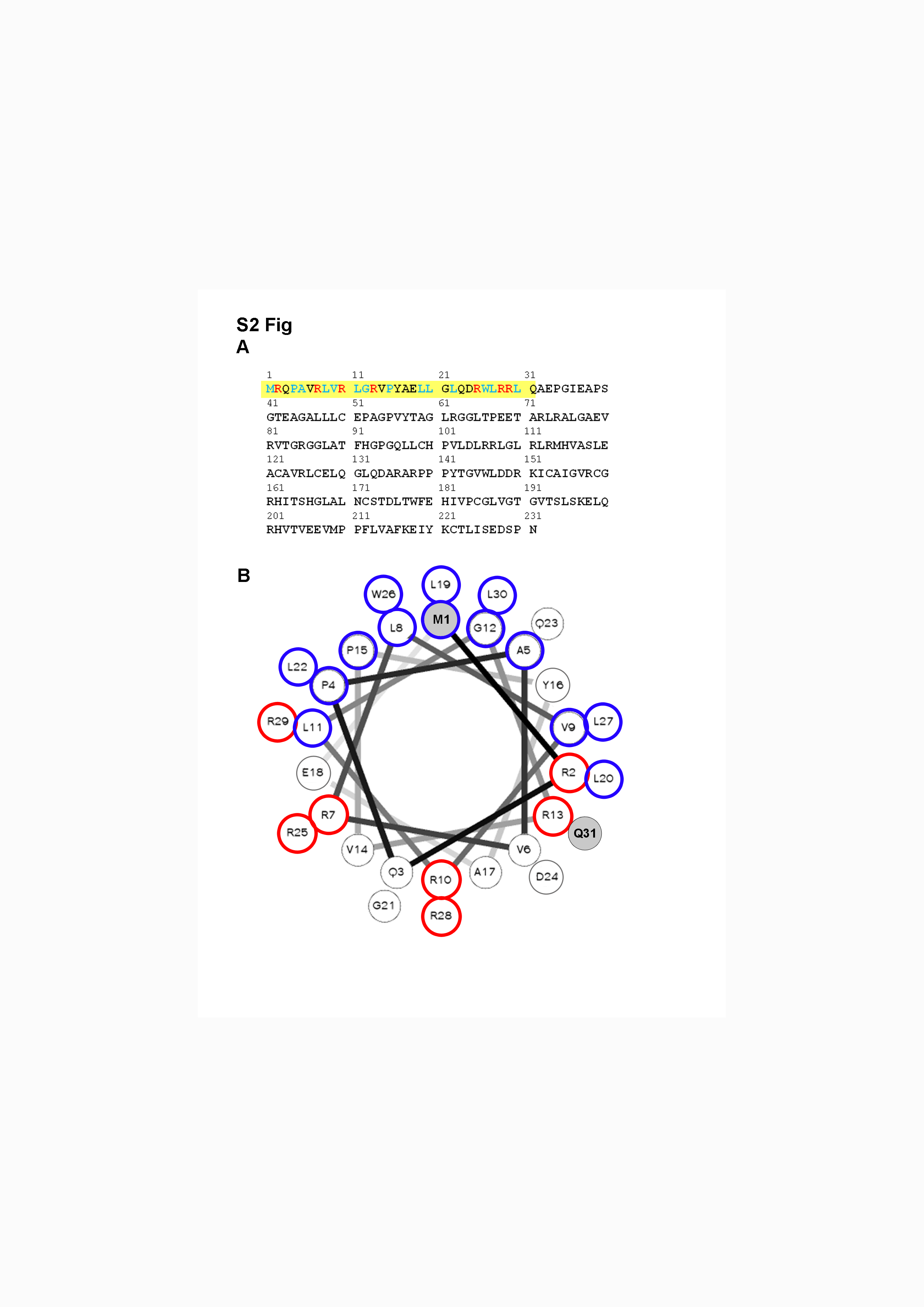

Supplement: S2 Fig — (A) Amino acid sequence of LIPT2. The mitochondrial targeting sequence (amino acids 1–31) is highlighted in yellow. (B) Helical wheel projection of the mitochondrial targeting sequence of LIPT2, with the first (M1) and the last (Q31) amino acids in grey circles. In (A) and (B), the amino acid residues that would bear a positive charge at physiological pH (Arg 2, 7, 10, 13, 25, 28 and 298) are indicated in red, and the non-polar residues (Met 1, Pro 4, Ala 5, Leu 8, Val 9, Leu 11, Gly 12, Pro 15, Leu 19, Leu 20, Leu 22, Trp 26, Leu 27 and Leu 30) are indicated in blue. The helical wheel projection was generated according to Gene Runner, 3.05 and Helical Wheel Projections, Version: Id: wheel.pl,v 1.4 2009-10-20 21:23:36 don Exp, http://rzlab.ucr.edu/scripts/wheel/wheel.cgi. (TIF) [file pone.0179591.s002.tif]

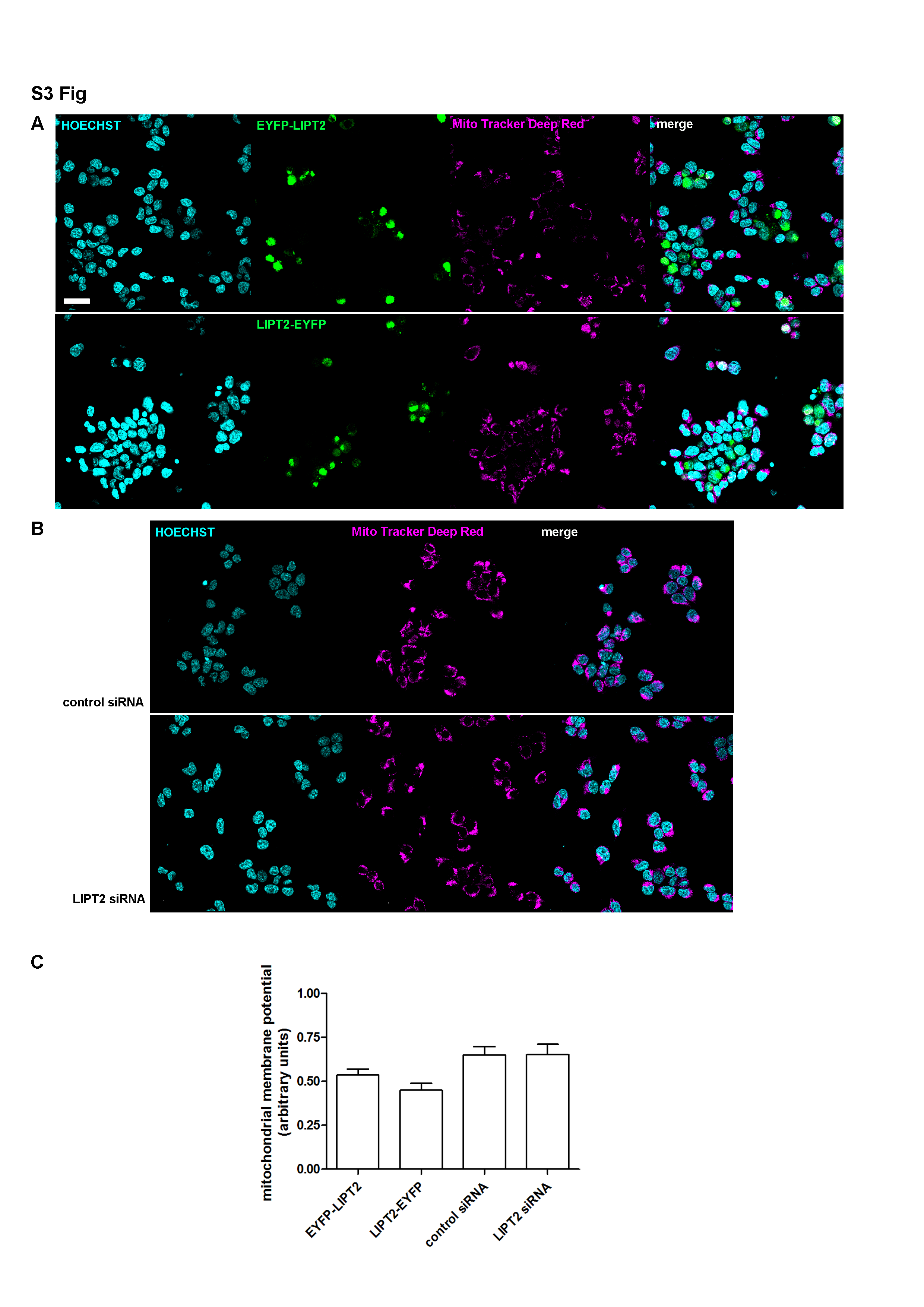

Supplement: S3 Fig — (A) From left to right: fluorescent signal of Hoechst (nucleus), EYFP, Mito Tracker Deep Red (mitochondrial potential) and corresponding merge image of HEK 293 Phoenix cells transfected for 48 hours with the indicated constructs. Scale bar: 30 μm. (B) From left to right: fluorescent signal of Hoechst (nucleus), Mito Tracker Deep Red (mitochondrial potential) and corresponding merge image of HEK 293 Phoenix cells transfected for 48 hours with control siRNA and LIPT2 siRNA#2. (C) Mitochondrial membrane potential normalized for the cell density. No statistically significant differences between groups were found, one-way ANOVA with Bonferroni’s post-test, n = 8. (n) indicates the number of imaging fields. (TIF) [file pone.0179591.s003.tif]

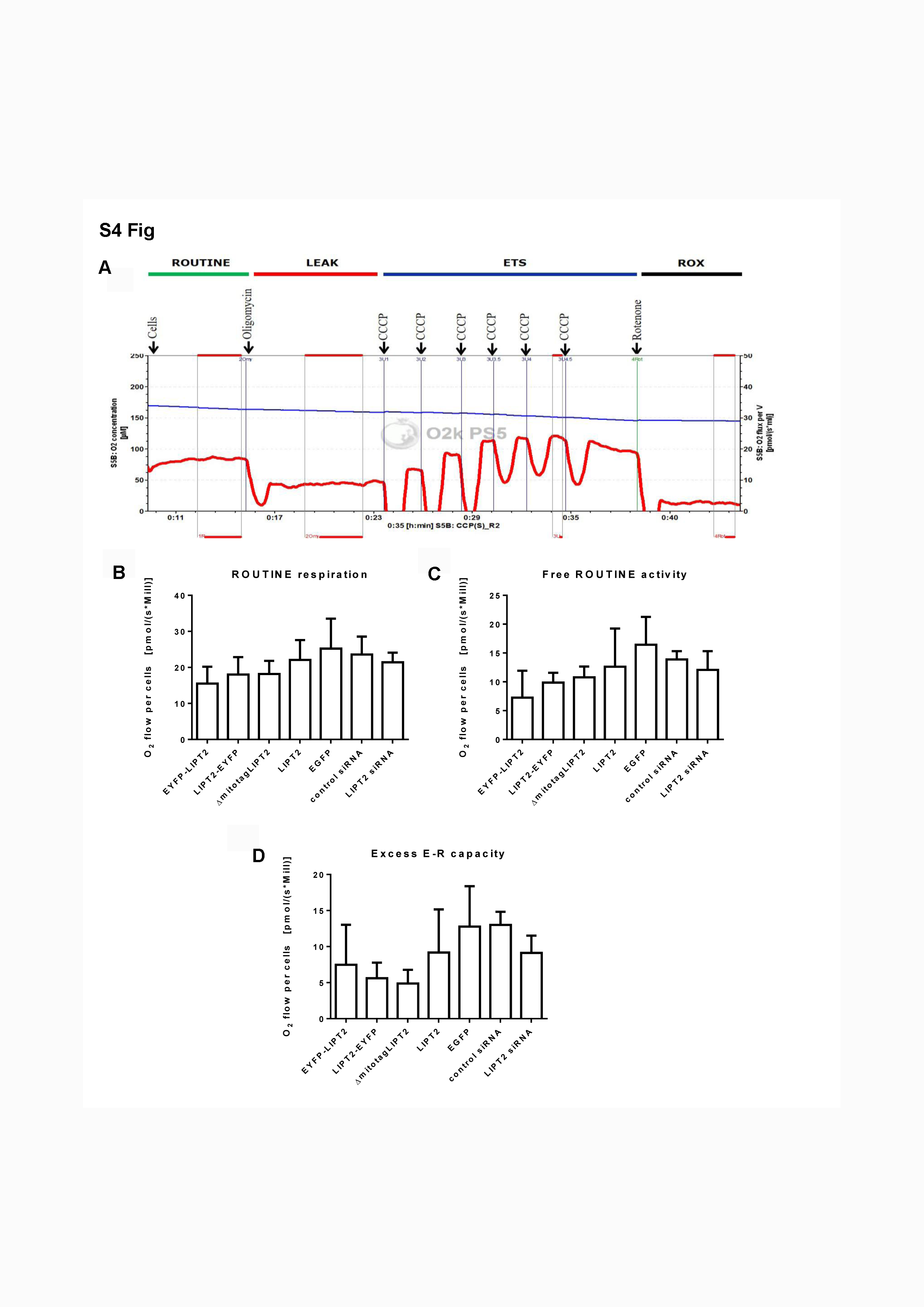

Supplement: S4 Fig — (A) Representative traces of the respiration in intact cells. The blue line represents the oxygen concentration, the red line the oxygen flux. A coupling control protocol was applied after adding the cells into the O2k-chambers. (B) ROUTINE respiration (R) reflecting cellular oxygen consumption under near-physiological conditions. In this state, cells experience neither saturating substrate conditions nor are they challenged by elevated cellular ATP demand. (C) Free ROUTINE activity, calculated as the difference between R and LEAK (L) respiration (R-L). The latter represents the component of respiration not related to ATP production, but supporting proton transfer compensating for dissipative proton fluxes across the inner mitochondrial membrane. (D) Respiratory excess capacity, representing the difference between electron transfer system (ETS) capacity and R. ETS reflects maximum respiratory activity observed when the limitation imposed by the oxidative phosphorylation system is removed by uncoupling respiration from oxidative phosphorylation. All data for oxygen flow are corrected for residual oxygen consumption (obtained after the inhibition of the complexes I and III by rotenone and antimycin A, respectively) and for cell viability. Cells were transfected for 48 hours with the indicated constructs. Data are means ± SEM of n = 4 cell cultures per experimental group. No statistically significant differences between groups were found, one-way ANOVA. (TIF) [file pone.0179591.s004.tif]

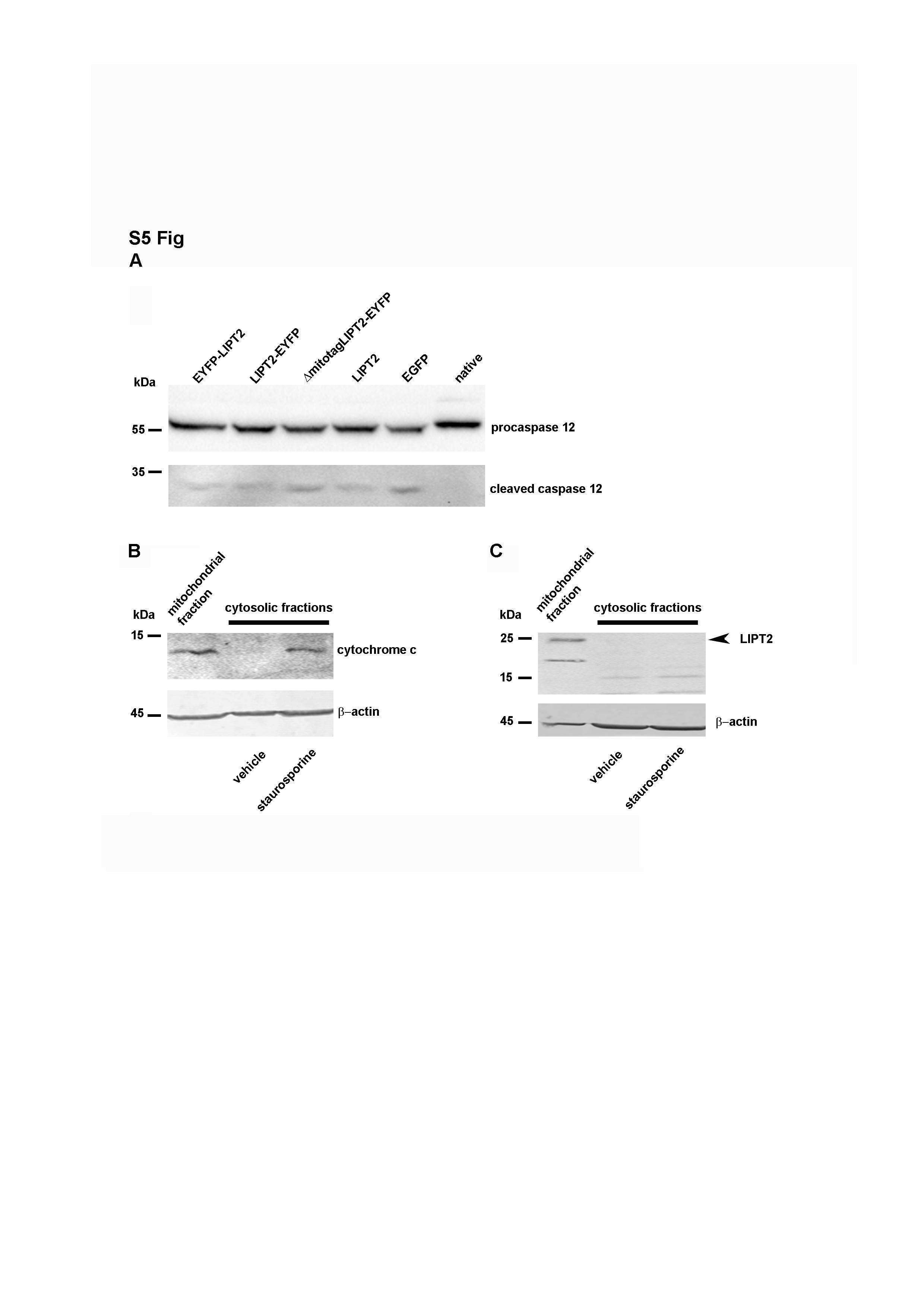

Supplement: S5 Fig — (A) HEK 293 Phoenix cells were transfected for 48 hours with the indicated constructs or left untransfected (native). Caspase-12 was immunodetected in whole cell lysates. Cleaved caspase-12 was retrieved in all samples from transfected cells, and not in native cells. The images are representative of 3 independent samples. (B) Untransfected HEK 293 Phoenix cells were treated either with 20 μM staurosporine or the vehicle for 4 hours. Cytochrome c could be detected in the mitochondrial fractions and in the cytosolic fraction of staurosporine-treated cells, but not in the cytosolic fraction of vehicle-treated cells. (C) In the same samples shown in (B), endogenous LIPT2 could only be detected in the mitochondrial fractions, but not in the cytosolic fractions of staurosporine or vehicle-treated cells. In (B) and (C), the housekeeping protein β-actin was immunodetected as a loading control. (TIF) [file pone.0179591.s005.tif]
