# Supplementary material for: Mis-targeting of the mitochondrial protein LIPT2 leads to apoptotic cell death
Source: PLoS One. 2017 Jun 19;12(6):e0179591. doi: 10.1371/journal.pone.0179591 (PMC5476274; doi:10.1371/journal.pone.0179591)
Supplement: S1 File — (DOCX) [file pone.0179591.s006.docx]

**Material and methods**

**Whole-field determination of mitochondrial membrane potential**

To determine mitochondrial membrane potential alterations, cells were transferred on round (3 cm diameter) poly-L-lysine-coated glass slides 32 hours post-transfection and imaged 48 hours post-transfection.

To stain the mitochondria and nuclei, living cells were washed thrice with HBSS, incubated for 30 minutes at room temperature with 1 µg/ml Hoechst (Sigma Aldrich) in HBSS in the presence of 300 nM Mito Tracker Deep Red for the last 15 minutes and washed again thrice with HBSS. In control experiments, the mitochondrial membrane potential collapse was induced with the uncoupling agent carbonylcyanide-4-trifluoromethoxyphenylhydrazone (FCCP, Sigma Aldrich; 20µM) added to the staining solution. The vehicle of FCCP was dimethyl sulfoxide (DMSO). Imaging was performed by sequential acquisition in HBSS at room temperature with a Leica TCS SP5II AOBS confocal microscope equipped with a HCX PL APO 63×/1.20 Lambda blue water immersion objective. Hoechst was excited with the 405 nm line of a diode laser and emission was detected in the 430-470 nm range; Mito Tracker Deep Red was excited at 633 nm (HeNe laser) and emission was detected in the 645-750 nm range. Fluorescence intensity of Mito Tracker Deep Red in the whole imaging field was normalized for the cells density in the same field. For this, fluorescence intensity of Mito Tracker Deep Red was expressed as levels of gray, subtracted for the background fluorescence and normalized for the background-subtracted fluorescence intensity of Hoechst.

**Mitochondrial function in intact cells**

Oxygen consumption of intact cells was measured at 37 ºC in the Oxygraph-2k (O2k) by high-resolution respirometry (OROBOROS Oxygraph, Innsbruck, Austria). Measurements were carried out in two chambers containing 2 ml cell culture medium (DMEM with 10% bovine serum albumin, 50 units/ml penicillin, and 50 µg/ml streptomycin) with continuous stirring (750 r.p.m.). Air calibration and instrumental background correction were employed and care was taken to maintain normoxic oxygen levels (~180 to 100 μM of oxygen) during the experiments. Oxygen consumption (pmol/(s·Mill cells)) was measured after addition of intact cells (0.41 ± 0.02 Mill cells/ml, final concentration) into the Oxygraph-2k chambers. A coupling control protocol (CCP), which induces different coupling control states, was used to evaluate the mitochondrial respiratory function (Fig. S5A). First, ROUTINE (*R*) respiration of the intact cells was measured. Then oligomycin A, an inhibitor of the ATP synthase, was added, providing LEAK (*L*) respiration. The *L* state is the non-phosphorylating resting state where oxygen flow is maintained mainly to compensate for the proton leak across the inner mitochondrial membrane. Next, stepwise titration (0.5 – 1 µM steps) of the uncoupler carbonyl cyanide m-chloro phenyl hydrazine (CCCP) up to maximum respiration was conducted to obtain respiration at the maximum capacity of the electron transfer system (ETS, *E*). Complex I was inhibited by using 1 µM of rotenone, blocking respiration in the intact cells. Finally, we obtained non-mitochondrial oxygen consumption (ROX) by inhibiting Complex III with antymicin A (2.5 μM). Data were acquired and analyzed with the software DatLab.
